# Supplementary material for: A set-theoretic definition of cell types with an algebraic structure on gene regulatory networks and application in annotation of RNA-seq data
Source: Stem Cell Reports. 2022 Nov 17;18(1):113–30. doi: 10.1016/j.stemcr.2022.10.015 (PMC9859932; doi:10.1016/j.stemcr.2022.10.015)
Supplement: Document S1. Figures S1–S7, Method details, and Supplemental experimental procedures [file mmc1.pdf]

Stem Cell Reports, Volume *18*

Supplemental Information

A set-theoretic definition of cell types with an algebraic structure on gene regulatory networks and application in annotation of RNA-seq data

Yuji Okano, Yoshitaka Kase, and Hideyuki Okano

Supplemental Experimental Procedures

Supplemental Methods

Tropical geometry and tropical semirings

Tropical geometry is considered over the classical tropical semifield $(\mathbb{R} \cup \{\infty\}, \oplus, \odot)$ defined as follows (Katz, 2017):

$$\forall a, b \in \mathbb{R} \cup \{\infty\}$$
$$a \oplus b = \min(a, b)$$
$$a \odot b = a + b$$

Note that a semifield $(K, +, \cdot)$ is a semiring such that $(K, \cdot)$ is an abelian group with the zero of the semifield K (i.e., an absorbing element of multiplication $a \in K$ such that $(K - \{a\}, \cdot)$ forms an abelian group (Mitchell and Sinutoke, 1982)); therefore, by definition, tropical semifields always fulfill the definition of a semiring. Other tropical analogs can be defined as well (Katz, 2017).

Commutative semiring

When $(\{0, 1, \infty\}, \oplus, \odot)$ is a commutative semiring (consider addition and multiplication to be defined as well as the tropical semiring), it fulfills all the following requirements (Pin, 2010; Zumbrägel and Zelmanov, 2008).

1. $\{0, 1, \infty\}$ is a commutative monoid for the addition (in this case, $\oplus$), with the neutral

element called $zero$ (in this case, ∞).

2. $\{0, 1, \infty\}$ is a monoid for the multiplication (in this case, $\odot$), with the neutral element called one (in this case, 0).

3. $\forall a, b, c, \in \{0, 1, \infty\}\ s.t.\ a(b \oplus c) = ab \oplus ac\ \wedge (a \oplus b)c = ac \oplus bc$ (i.e., multiplication is distributive over addition).

4. $\forall a \in \{0, 1, \infty\}, \exists! \infty \in \{0, 1, \infty\}, a \oplus \varepsilon = \varepsilon \oplus a = a\ \ s.t.\ a \odot \varepsilon = \varepsilon \odot a = \varepsilon$ (i.e., the neutral element for the addition is an absorbing element for the multiplication).

5. Multiplication is commutative.

Quotient semiring and natural homomorphism

Suppose $\forall (R, +, \cdot), (S, +, \cdot): Semirings, \forall f: R \rightarrow S\ \ s.t.\ \forall r_1, r_2 \in R, f(r_1 + r_2) = f(r_1) + f(r_2) \wedge f(r_1 r_2) = f(r_1) \cdot f(r_2)$, where f is the map that preserves the semiring operations and the zero element (hence defined as a semiring homomorphism) (Zumbrägel and Zelmanov, 2008). Moreover, we considered an equivalence relation $\sim$ on a semiring $(R, +, \cdot)$ as congruence where the notation $r_1 \sim r_2$ implies $r_1 + s \sim r_2 + s \wedge sr_1 \sim sr_2 \wedge r_1 s \sim r_2 s$ for all $r_1, r_2, s \in R$ (Zumbrägel and Zelmanov, 2008). When these definitions for a homomorphism and congruence are given, we can define the same operations ($+$ and $\cdot$) as R on its set of equivalence classes $R/{\sim} = \{[r_1] | r_1 \in R\}$

by $[r_1] + [r_2] \coloneqq [r_1 + r_2]$ and $[r_1] \cdot [r_2] \coloneqq [r_1 r_2]$ for all $r_1, r_2 \in R$ (an equivalent class is denoted as $[r_1] \coloneqq \{r_2 \in R | r_1 \sim r_2\}$ and $(R/\sim, +, \cdot)$ is called the quotient semiring). In addition, the map $\pi: R \to R/\sim$ used to generate the quotient semiring of R always satisfies congruence $\sim$ on R, because we define $r_1 \sim r_2$ if and only if $\pi(r_1) = \pi(r_2)$ for all $r_1, r_2 \in R$; thus, π is called the natural homomorphism $R \to R/\sim$ (Zumbrägel and Zelmanov, 2008).

Ideal on a semiring

The definition of an ideal I_R on a semiring $(R, +, \cdot)$ is given as follows (Allen, 1969):

$$\forall I_R \subset R, \forall i, j \in I_R, \forall r \in R \;\; s.t. \;\; i + j \in I_R \;\wedge\; r \cdot i \in I_R \;\wedge\; i \cdot r \in I_R.$$

When I_R is an ideal of R, $[r] = r + I_R \coloneqq \{r + i | i \in I_R\}$ for all $r \in R$. Hence, the design of the ideals determines the configuration of the equivalent relation ($\forall r \in R, \forall i \in I_R \; s.t. \; r \sim r + i$) and corresponding equivalent classes. Note that $(R/I_R, +, \cdot)$ is a quotient semiring, where $R/I_R = \{[r] | r \in R\}$.

Hamming Distance

The Hamming distance is frequently used to quantify the difference between two bitstrings of the same dimension (i.e., length) (Bookstein et al., 2002). It is defined as the

number of mismatches in the corresponding bits of the two strings. The three strings 100010, 010010, and 001010 all differ from each other by a Hamming distance of 2 (Bookstein et al., 2002).

Topological space and open ball

A topology on a set X is defined as a family $\mathcal{O}$ of subsets of X (called open sets) where $\mathcal{O}$ satisfies the following:

1. $\emptyset \subset \mathcal{O} \wedge X \subset \mathcal{O}$.

2. $\forall \Lambda\text{: Set}, \forall \lambda \in \Lambda,\ o_\lambda \in \mathcal{O}\ s.t. \bigcup_{\lambda \in \Lambda} o_\lambda \in \mathcal{O}$.

3. $\forall O_1, O_2 \in \mathcal{O}\ s.t. O_1 \cap O_2 \in \mathcal{O}$.

When a set is equipped with a topology, it is called a topological space. In terms of X and $\mathcal{O}$, $(X, \mathcal{O})$ is a topological space (Manetti, 2015).

The definition of the open ball requires a distance function; hence, we verify this here. A distance on a set X is a function $d: X \times X \to \mathbb{R}$ that satisfies the following criteria:

1. $\forall x, y \in X, d(x, y) \geq 0 \wedge d(x, y) = 0 \Leftrightarrow x = y$.

2. $\forall x, y \in X, d(x, y) = d(y, x)$.

3. $\forall x, y, z \in X, d(x, y) + d(y, z) \geq d(x, z)$.

Note that a metric space is a pair (X, d) consisting of a set X and a distance d on X. The topology of the metric space (called the metric topology) is determined by the distance and described using open balls. An open ball $B(x, r)$ centered at x with radius r is a subset of the metric space defined with parameters $x, r \in \mathbb{R}$ as

$$B(x, r) \coloneqq \{y \in X | d(x, y) < r\}.$$

Note that $B_r(x)$ is an equivalent notation, which is being curried about r. Given the definition of an open ball, the metric topology $\mathcal{T}$ is defined as follows:

$$\mathcal{T} \coloneqq \{T \subset X | \forall x \in T, \exists r \in \mathbb{R}_{>0}\ s.t. B(x, r) \subset T\},$$

where $\mathbb{R}_{>0}$ is a positive subset of real numbers. The definition of metric topology satisfies the definition of topology (Manetti, 2015).

Quasi-pseudo-metric, pseudo-metric, and topology of pseudo-metric space

A quasi-pseudo-metric p is a mapping $p: X \times X \to \mathbb{R}$, where X is a set and p satisfies the following (Kũnzi, 1992):

1. $\forall x, y \in X, p(x, y) \geq 0.$

2. $\forall x \in X, p(x, x) = 0.$

3. $\forall x, y, z \in X, p(x, y) + p(y, x) \geq p(x, z).$

A pseudo-metric ρ is a mapping $\rho: X \times X \to \mathbb{R}$, where X is a set and p satisfies

the following (Herrlich and Keremedis, 2015):

1. $\forall x, y \in X, \rho(x, y) \geq 0 \land \rho(x, x) = 0$.

2. $\forall x, y \in X, \rho(x, y) = \rho(y, x)$.

3. $\forall x, y, z \in X, \rho(x, y) + \rho(y, z) \geq \rho(x, z)$.

Given the definition of pseudo-metric, (X, ρ) is a pseudo-metric space. Pseudo-metric topology is also generated by an open ball $D(x, \epsilon)$, defined as

$$D(x, \epsilon) = \{y \in X | \rho(x, y) < \epsilon\} .$$

When we introduce an equivalence relation $\sim$ on X (given by $x \sim y$ if and only if $\rho(x, y) = 0$), the quotient space (X^*, ρ^*) of (X, ρ) is a metric space because the metric reflection is introduced by $\sim$, where X^* is the set of all equivalent classes in X and $\rho^*: X^* \times X^* \to \mathbb{R}$ is given by the following; meanwhile, $[x]$ is the equivalent class of $x \in X$ (Herrlich and Keremedis, 2015), such that

$$\rho^*([x], [y]) = \rho(x, y).$$

Method details

Under-sampling

Random under-sampling was performed to resolve the class imbalance in m1_10x. Samples were stratified according to the metadata; then, resampling was performed to make the sample sizes of all cell types even numbers. The resampled data were used for GBDT, factor analysis, DEG-based manual annotation, and GRN-based annotation. The code was implemented in Python packages (Imbalanced-learn, Numpy, and Pandas).

Data splitting

To prevent data leakage, groups were generated for the "GroupShuffleSplit" method in Scikit-learn. The expression matrix for the 90 marker genes in the resampled m1_10x were converted into a matrix with lower dimensionality, via principal component analysis (PCA); meanwhile, the number of components was automatically determined (Minka, 2001), then, samples were classified into 500 clusters using k-means algorithm. Next, the resampled data were randomly split into training/test data (4:1) using the GroupShuffleSplit method. The random seed was fixed throughout the process. The

code was implemented using a Python package (Scikit-learn).

Factor analysis

Factor analysis using quartimin rotation was performed. The initial number of factors was determined according to parallel analysis with null models of randomly permutated data matrices. After quartimin rotation, if the model included factors with maximum absolute factor loading values smaller than 0.5, parallel analysis was performed on the factor score matrices and a reduced number of factors was obtained. These processes were iteratively performed whilst factors with small loadings were retained in the model. The code was implemented using Python packages (Factor_analyzer, Matplotlib, Numpy, Pandas, and Seaborn).

K-means clustering

K-means clustering was performed. The optimal number of clusters was determined by the mean silhouette scores. The random seed was fixed throughout the process. The code was implemented in Python packages (Matplotlib, Pandas, Scikit-learn, and Seaborn).

SNN clustering

In the DEG-based annotation, clustering was performed using the "FindClusters" method with resolution of 0.8. Other arguments were fixed as default values. The code was performed using an R package (Seurat).

Identification of variable features and DEGs

For gw9–gw12, 2000 variable features were identified for each using "FindVariableFeatures" method with default arguments. DEGs were identified for each cluster using "FindAllMakers" method. The threshold for log FC was set to 0.25. The code was implemented using an R package (Seurat).

Dimensionality reduction (truncated SVD and UMAP)

The data dimensionality was determined via parallel analysis for the explained variance calculated using truncated SVD. The data were sent for further analysis once the decomposed matrices had been cropped to match the dimensionality of the parallel analysis. For gw9–gw12, the matrices of lower dimensionality were embedded into the UMAP manifold using the "RunUMAP" method. For m1_10x, after PCA dimensionality reduction, the UMAP method was used to embed the matrix. The codes were

implemented in R packages (Seurat, Dplyr, and Jsonlite) and Python packages (Matplotlib, Pandas, Scikit-learn, Scipy, Seaborn, and Umap-learn).

GBDT

GBDT with L1 and L2 regularizations was selected as an ML model for multiclass classification. To reduce the computational costs, the 1000 genes with the highest S.D. amongst the group-wise mean values were selected as features. Hyperparameter tuning was performed using five-fold cross validation with logarithmic loss chosen as the objective function. The FI was calculated in each fold. The prediction performance of the model with optimized hyperparameters was verified on the test data according to multiple indices (AUC of ROC curve, AP, macro average of AUC, and micro average of AP). To ensure that the important features adequately explained the classification, a new GBDT model containing the important features was constructed via the same process. The code was implemented using Python packages (LightGBM, Matplolib, Numpy, Optuna, Pandas, Scikit-learn, and Seaborn).

PC algorithm and GRN construction

The expression matrix of the genes exhibited high communalities in the factor analysis

for the resampled m1_10x and important features of the GBDT model were selected. DAGs were obtained using the PC algorithm with a test of uncorrelation in terms of Pearson's correlation coefficient (significance level: 0.01). To convert the DAGs into undirected GRNs (without ε), the directionalities of the edges were neglected. The edge information was converted into matrices by overwriting the corresponding elements in an $n \times n$ identity matrix with 1 if the edge existed in the GRN (where n is the number of vertexes). The code was implemented using Python packages (Numpy, Pandas, and Pgmpy).

Visualization of GRN-based annotation

The values of d^* were calculated from the matrices that storing GRN information. The cardinalities of the eigen-cascades were calculated as the sum of all elements in the matrix, and the intersections of the eigen-cascades were calculated as Hadamard products. Note that the diagonal components correspond to edges from ε to all vertexes. The code was implemented using Python packages (Matplolib, Numpy, and Pandas).

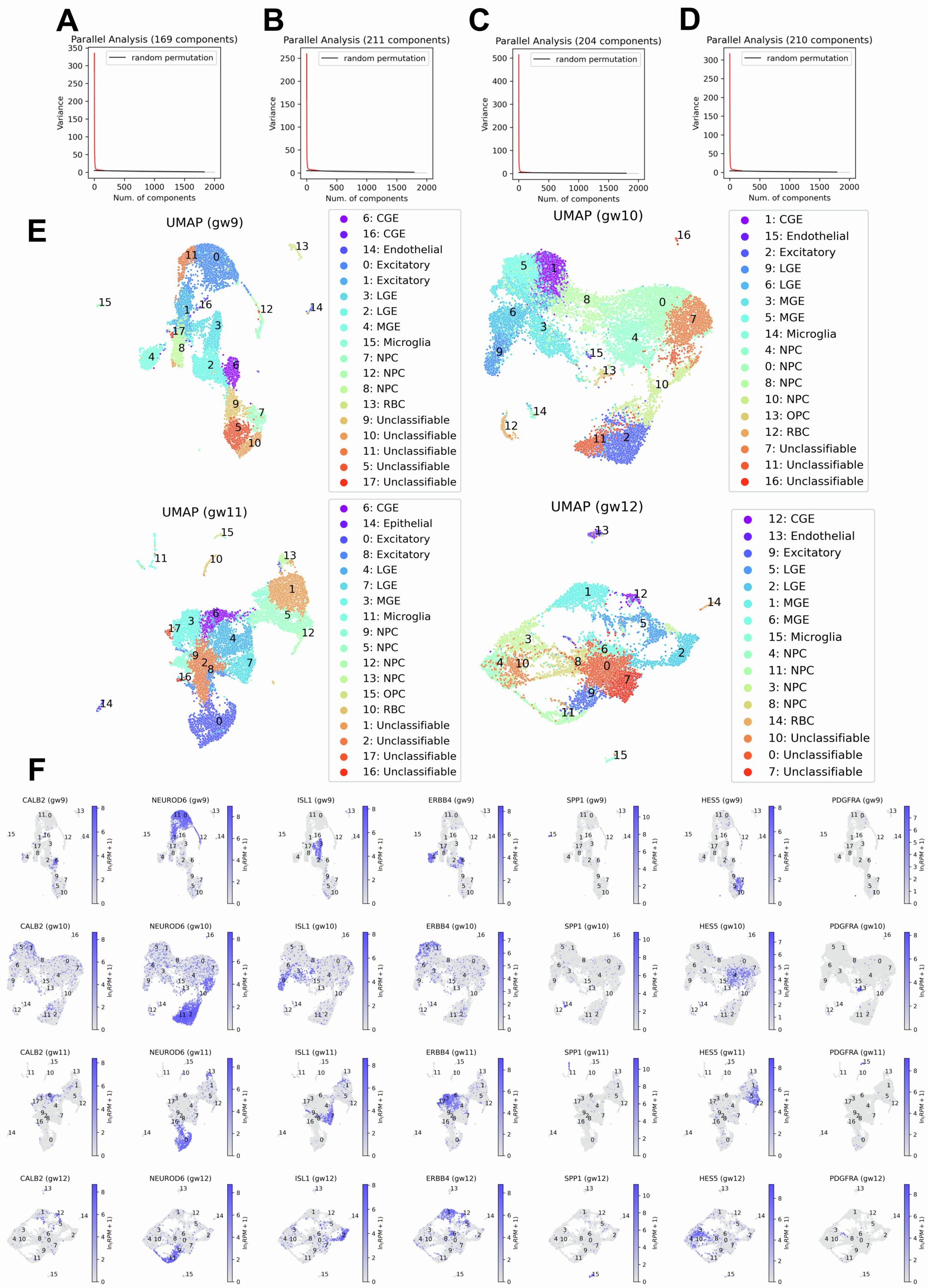
A
Parallel Analysis (169 components)
B
Parallel Analysis (211 components)
C
Parallel Analysis (204 components)
D
Parallel Analysis (210 components)
random permutation
Variance
Num. of components
E
UMAP (gw9)
6: CGE
16: CGE
14: Endothelial
0: Excitatory
1: Excitatory
3: LGE
2: LGE
4: MGE
15: Microglia
7: NPC
12: NPC
8: NPC
13: RBC
9: Unclassifiable
10: Unclassifiable
11: Unclassifiable
5: Unclassifiable
17: Unclassifiable
UMAP (gw10)
1: CGE
15: Endothelial
2: Excitatory
9: LGE
6: LGE
3: MGE
5: MGE
14: Microglia
4: NPC
0: NPC
8: NPC
10: NPC
13: OPC
12: RBC
7: Unclassifiable
11: Unclassifiable
16: Unclassifiable
UMAP (gw11)
6: CGE
14: Epithelial
0: Excitatory
8: Excitatory
4: LGE
7: LGE
3: MGE
11: Microglia
9: NPC
5: NPC
12: NPC
13: NPC
15: OPC
10: RBC
1: Unclassifiable
2: Unclassifiable
17: Unclassifiable
16: Unclassifiable
UMAP (gw12)
12: CGE
13: Endothelial
9: Excitatory
5: LGE
2: LGE
1: MGE
6: MGE
15: Microglia
4: NPC
11: NPC
3: NPC
8: NPC
14: RBC
10: Unclassifiable
0: Unclassifiable
7: Unclassifiable
F
CALB2 (gw9)
NEUROD6 (gw9)
ISL1 (gw9)
ERBB4 (gw9)
SPP1 (gw9)
HES5 (gw9)
PDGFRA (gw9)
CALB2 (gw10)
NEUROD6 (gw10)
ISL1 (gw10)
ERBB4 (gw10)
SPP1 (gw10)
HES5 (gw10)
PDGFRA (gw10)
CALB2 (gw11)
NEUROD6 (gw11)
ISL1 (gw11)
ERBB4 (gw11)
SPP1 (gw11)
HES5 (gw11)
PDGFRA (gw11)
CALB2 (gw12)
NEUROD6 (gw12)
ISL1 (gw12)
ERBB4 (gw12)
SPP1 (gw12)
HES5 (gw12)
PDGFRA (gw12)
ln(RPM + 1)

Figure S1

Figure S1. DEG-based annotation on GSE165388

(A–D) The parallel analyses used to determine the dimensionalities of variable features in gw9–gw12.

(E) Results of SNN clustering in UMAP. The clusters refer to the DEGs.

(F) The expression patterns of representative marker genes for their respective cell types.

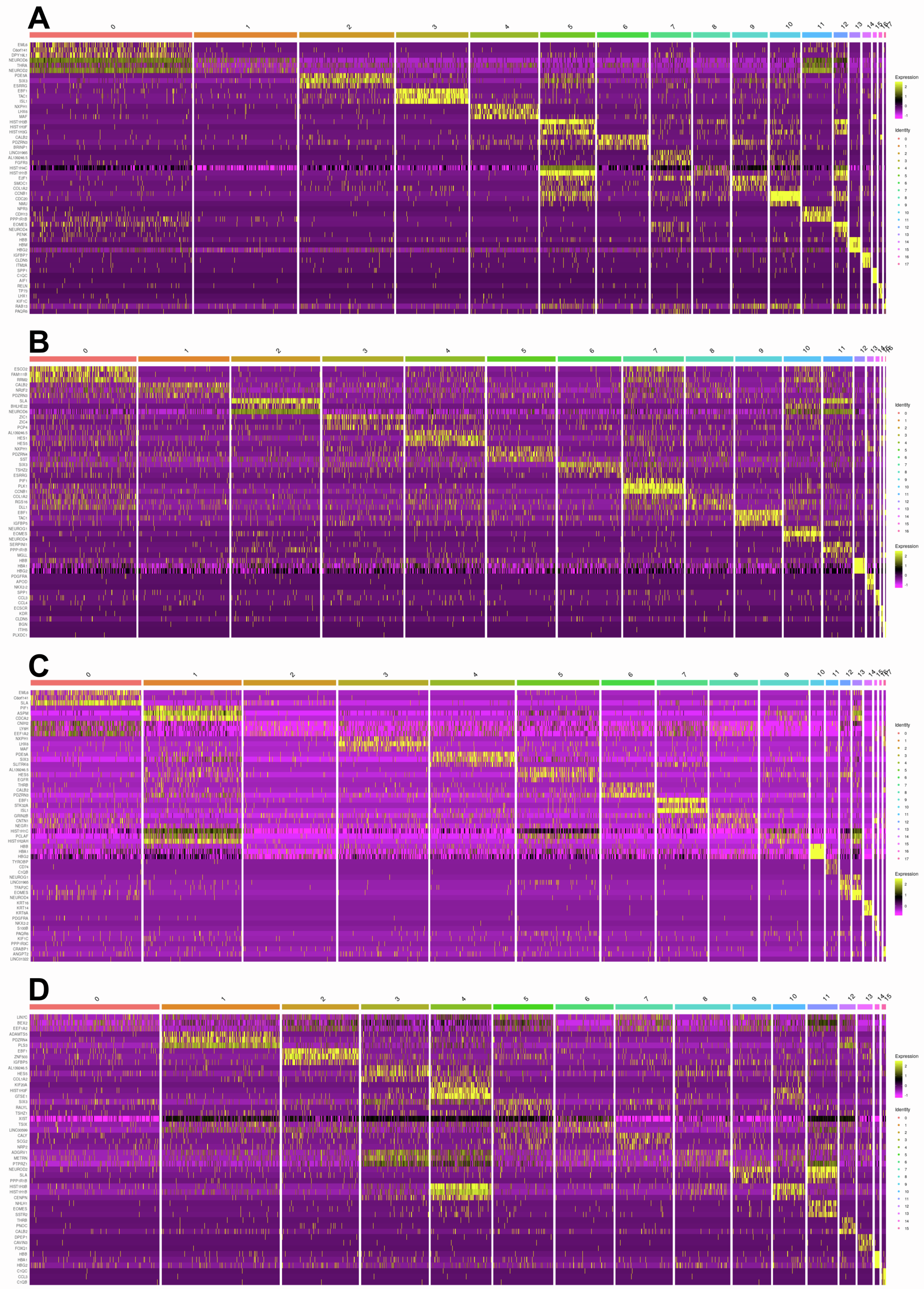

Figure S2

Figure S2. The expression patterns of DEGs, which are inconsistent even for the clusters under the same name.

(A–D) Heatmaps of the DEGs for their respective clusters. The Wilcoxon rank-sum test was performed to identify DEGs in (A) gw9, (B) gw10, (C) gw11, and (D) gw12. The top three genes with the largest log FC values are shown for each cluster.

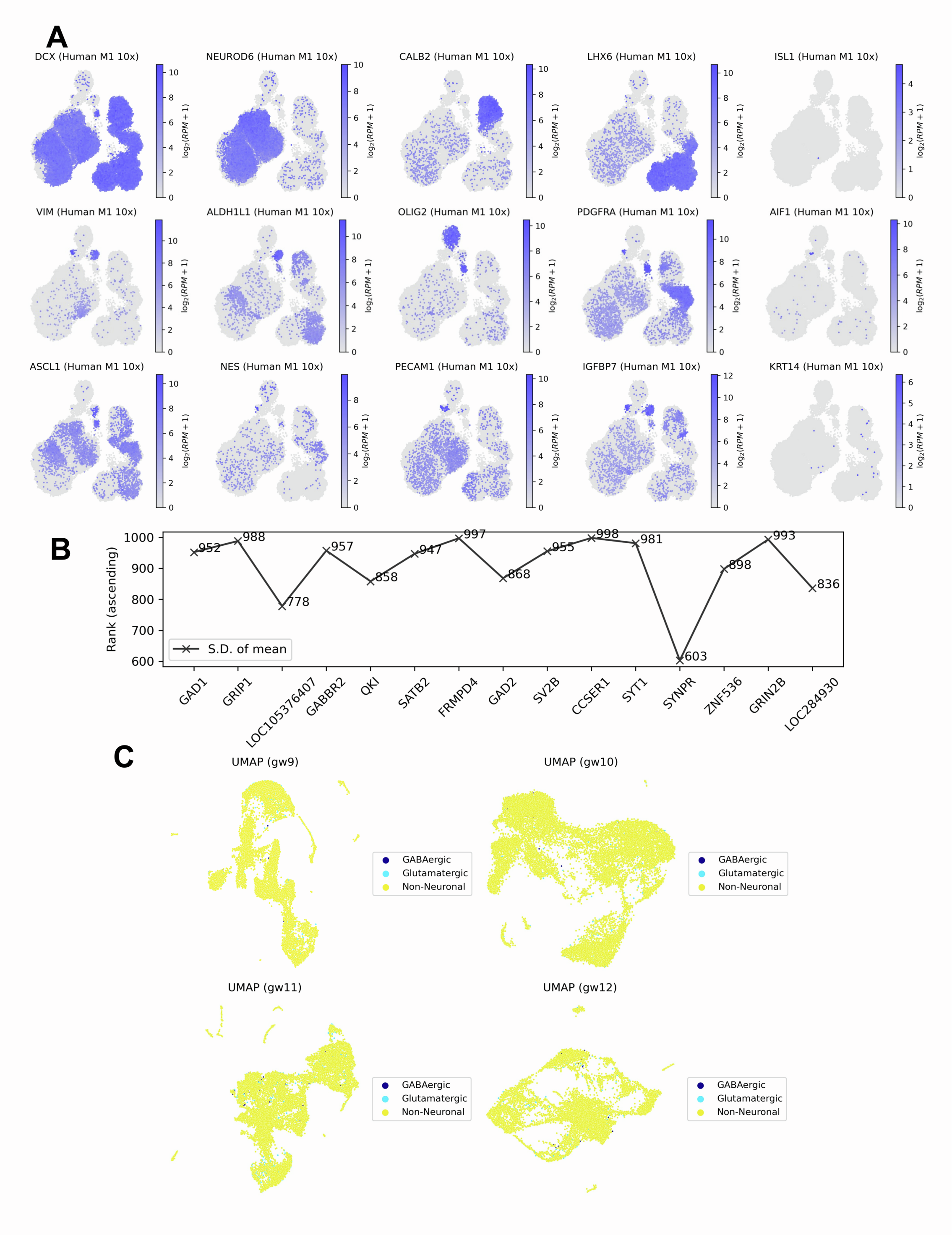
A
DCX (Human M1 10x)
NEUROD6 (Human M1 10x)
CALB2 (Human M1 10x)
LHX6 (Human M1 10x)
ISL1 (Human M1 10x)
VIM (Human M1 10x)
ALDH1L1 (Human M1 10x)
OLIG2 (Human M1 10x)
PDGFRA (Human M1 10x)
AIF1 (Human M1 10x)
ASCL1 (Human M1 10x)
NES (Human M1 10x)
PECAM1 (Human M1 10x)
IGFBP7 (Human M1 10x)
KRT14 (Human M1 10x)
log2(RPM + 1)
B
Rank (ascending)
S.D. of mean
GAD1 GRIP1 LOC105376407 GABBR2 QKI SATB2 FRMPD4 GAD2 SV2B CCSER1 SYT1 SYNPR ZNF536 GRIN2B LOC284930
952 988 778 957 858 947 997 868 955 998 981 603 898 993 836
C
UMAP (gw9)
UMAP (gw10)
UMAP (gw11)
UMAP (gw12)
GABAergic
Glutamatergic
Non-Neuronal

Figure S3

Figure S3. Supplemental information for the m1_10x and the GBDT model.

(A) Expression patterns of representative marker genes for their respective cell types.

(B) The relation between S.D. of group-wise mean values and FI for the top 15 genes (in terms of FI) in the GBDT model.

(C) Automatic annotation results obtained using the GBDT model. Our model was not designed for automatic annotation; hence, prediction performance was poor. The datasets from GSE165388 were converted into $\log_2$(RPM+1), to adjust the data scales in m1_10x before prediction.

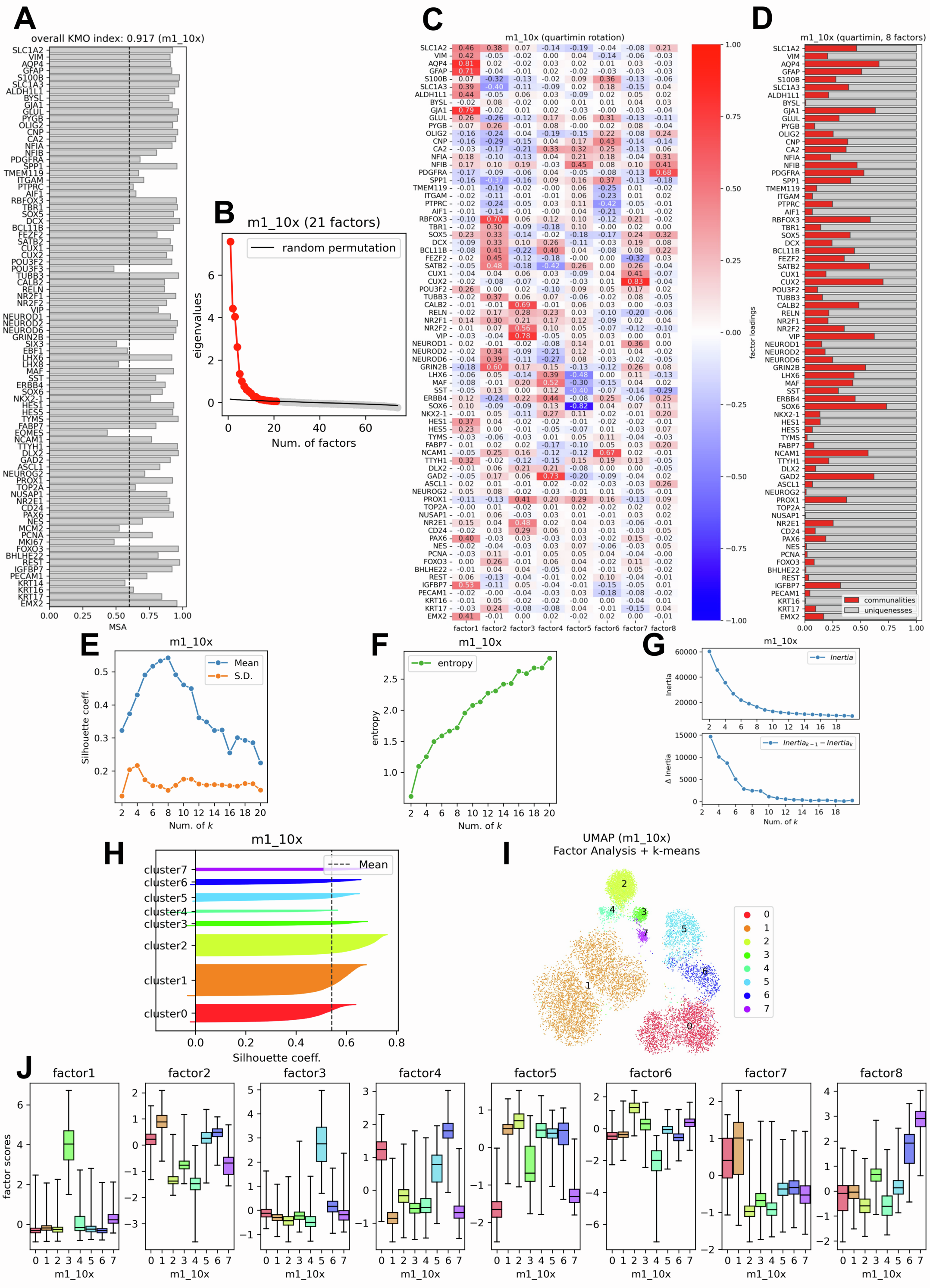
A
overall KMO index: 0.917 (m1_10x)
MSA
B
m1_10x (21 factors)
random permutation
eigenvalues
Num. of factors
C
m1_10x (quartimin rotation)
factor loadings
factor1 factor2 factor3 factor4 factor5 factor6 factor7 factor8
D
m1_10x (quartimin, 8 factors)
communalities
uniquenesses
E
m1_10x
Mean
S.D.
Silhouette coeff.
Num. of k
F
m1_10x
entropy
Num. of k
G
m1_10x
Inertia
Δ Inertia
Num. of k
H
m1_10x
Mean
cluster7
cluster6
cluster5
cluster4
cluster3
cluster2
cluster1
cluster0
Silhouette coeff.
I
UMAP (m1_10x)
Factor Analysis + k-means
J
factor1
factor2
factor3
factor4
factor5
factor6
factor7
factor8
factor scores
m1_10x

Figure S4

Figure S4. Factor analysis + k-means clustering in m1_10x.

(A) Overall KMO index and MSA values in each variable.

(B) Parallel analysis used to determine the number of factors.

(C) Heatmaps of factor loadings after eliminating factors with maximum loadings smaller than 0.5. Quartimin rotation was performed for all steps.

(D) Communalities and the uniquenesses for each variable in the model.

(E) Transition of the mean and S.D. of the silhouette coefficients.

(F) Transition of entropies for categorical distributions in which parameters were point-estimated using the sample size ratios of clusters.

(G) Elbow plot and the difference of inertia ($\Delta Inertia_k \coloneqq Inertia_{k-1} - Inertia_k$).

(H) Silhouette plot in the optimal k value.

(I) Result of k-means clustering in UMAP.

(J) Coordinates of clusters (corresponding to factors) in each axis.

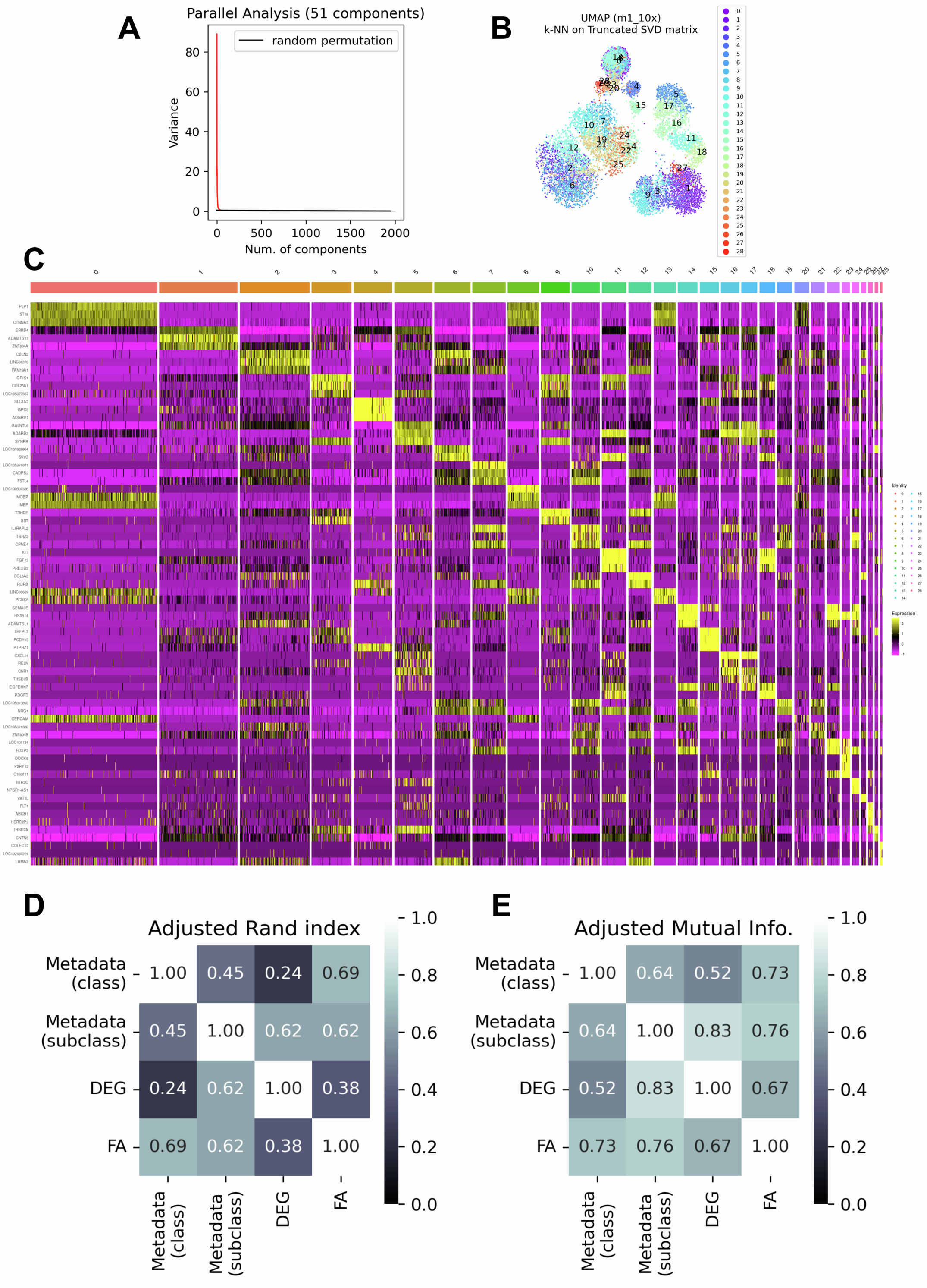
A
Parallel Analysis (51 components)
random permutation
Variance
Num. of components
B
UMAP (m1_10x)
k-NN on Truncated SVD matrix
C
D
Adjusted Rand index
Metadata (class)
Metadata (subclass)
DEG
FA
1.00 0.45 0.24 0.69
0.45 1.00 0.62 0.62
0.24 0.62 1.00 0.38
0.69 0.62 0.38 1.00
E
Adjusted Mutual Info.
Metadata (class)
Metadata (subclass)
DEG
FA
1.00 0.64 0.52 0.73
0.64 1.00 0.83 0.76
0.52 0.83 1.00 0.67
0.73 0.76 0.67 1.00

Figure S5

Figure S5. Conventional workflow for scRNA-seq data processing in m1_10x.

(A) Parallel analyses used to determine the dimensionalities of variable features in m1_10x.

(B) Results of SNN clustering in UMAP.

(C) Heatmaps of the DEGs for respective clusters. The Wilcoxon Rank Sum test was performed to identify DEGs in m1_10x. The top three genes with the largest log FC values were shown for each cluster.

(D–E) Performance comparison between the conventional method and our proposed method (factor analysis + k-means). (D) ARI and (E) AMI were calculated from comparisons of the class labels (cell type information) and subclass labels in the metadata of m1_10x.

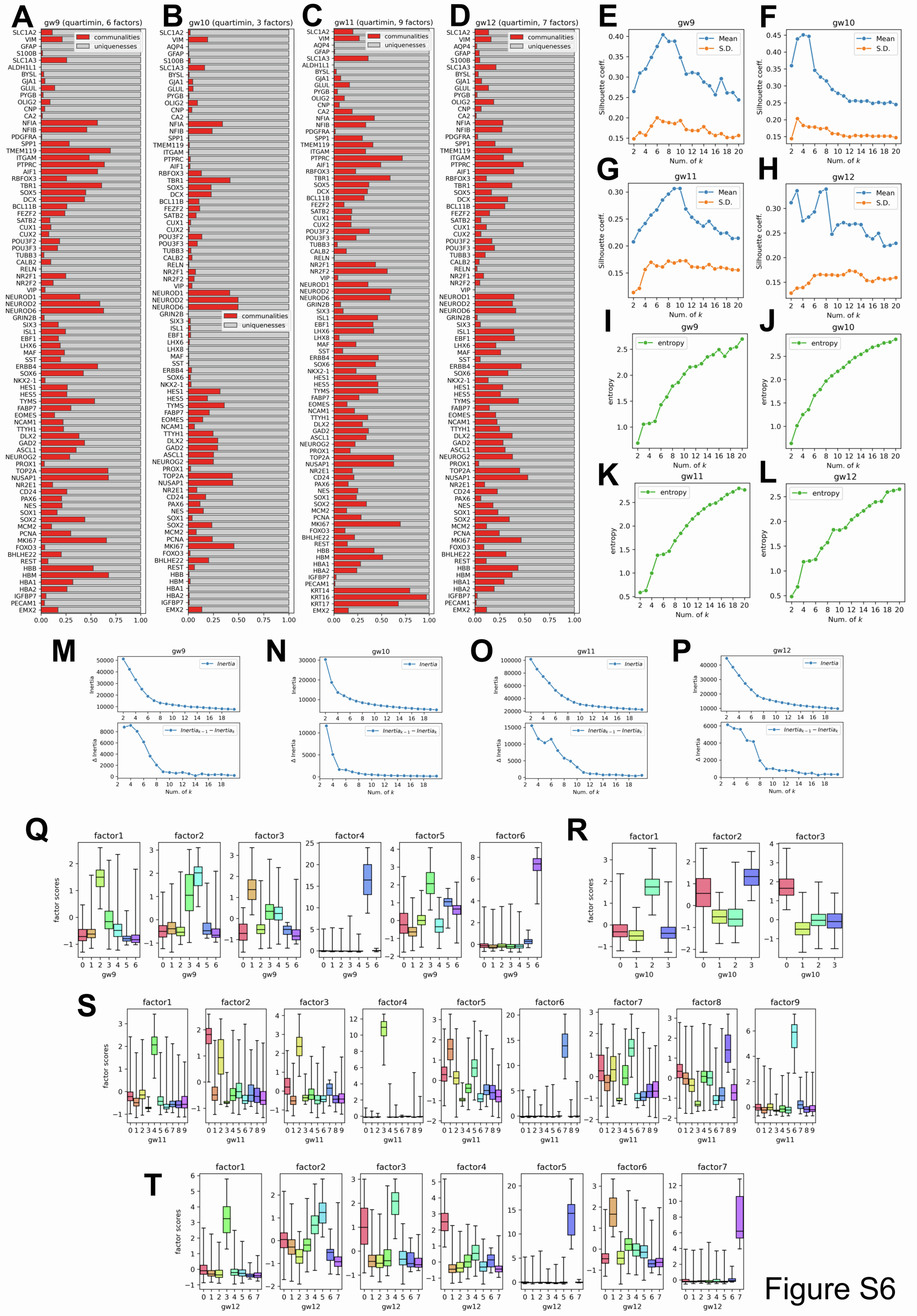
A
gw9 (quartimin, 6 factors)
B
gw10 (quartimin, 3 factors)
C
gw11 (quartimin, 9 factors)
D
gw12 (quartimin, 7 factors)
communalities
uniquenesses
E
gw9
F
gw10
G
gw11
H
gw12
Mean
S.D.
Silhouette coeff.
Num. of k
I
gw9
J
gw10
K
gw11
L
gw12
entropy
M
gw9
N
gw10
O
gw11
P
gw12
Inertia
Δ Inertia
Q
R
S
T
factor1
factor2
factor3
factor4
factor5
factor6
factor7
factor8
factor9
factor scores

Figure S6

Figure S6. Supplemental information for factor analysis + k-means clustering in GSE265388.

(A–D) The communalities and the uniquenesses for each variable in the models.

(E–H) Transition of the mean and S.D. for the silhouette coefficients.

(I–L) Transition of entropies for categorical distributions in which parameters were point-estimated using the sample size ratios of clusters.

(M–P) The elbow plots and the differences of inertia ($\Delta Inertia_k \coloneqq Inertia_{k-1} - Inertia_k$).

(Q–T) Coordinates of clusters (corresponding to factors) in each axis.

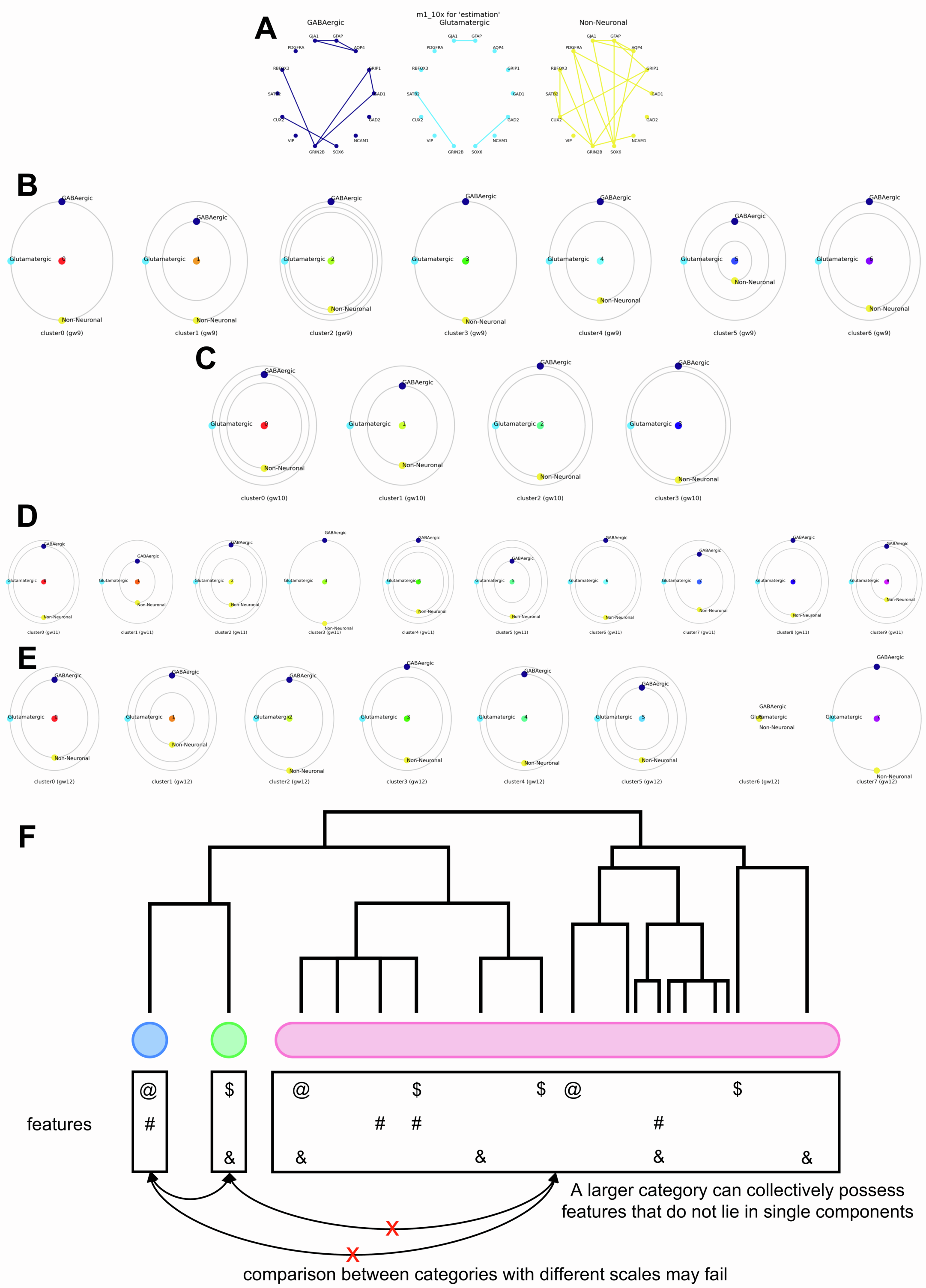
A
m1_10x for 'estimation'
GABAergic
Glutamatergic
Non-Neuronal
B
cluster0 (gw9)
cluster1 (gw9)
cluster2 (gw9)
cluster3 (gw9)
cluster4 (gw9)
cluster5 (gw9)
cluster6 (gw9)
C
cluster0 (gw10)
cluster1 (gw10)
cluster2 (gw10)
cluster3 (gw10)
D
E
cluster0 (gw12)
cluster1 (gw12)
cluster2 (gw12)
cluster3 (gw12)
cluster4 (gw12)
cluster5 (gw12)
cluster6 (gw12)
cluster7 (gw12)
F
features
A larger category can collectively possess
features that do not lie in single components
comparison between categories with different scales may fail

Figure S7

Figure S7. Supplemental information for GRN-based annotation in GSE165388.

(A) GRNs of cell types in m1_10x to be used in estimation (as referential data).

(B–E) Planet plots to show the estimation results.

(F) Demonstration that comparisons between differently scaled categories can fail.
