# Supplementary material for: A set-theoretic definition of cell types with an algebraic structure on gene regulatory networks and application in annotation of RNA-seq data
Source: Stem Cell Reports. 2022 Nov 17;18(1):113–30. doi: 10.1016/j.stemcr.2022.10.015 (PMC9859932; doi:10.1016/j.stemcr.2022.10.015)
Supplement: Document S2. Article plus supplemental information [file mmc2.pdf]

Stem Cell Reports
Article

OPEN ACCESS

A set-theoretic definition of cell types with an algebraic structure on gene regulatory networks and application in annotation of RNA-seq data

Yuji Okano,[1] Yoshitaka Kase,[1,2] and Hideyuki Okano[1,*]
[1]Department of Physiology, Keio University School of Medicine, 35, Shinanomachi, Shinjuku-ku, Tokyo 160-8582, Japan
[2]Senior author
*Correspondence: hidokano@keio.jp
https://doi.org/10.1016/j.stemcr.2022.10.015

SUMMARY

The emergence of single-cell RNA sequencing (RNA-seq) has radically changed the observation of cellular diversity. Although annotations of RNA-seq data require preserved properties among cells of an identity, annotations using conventional methods have not been able to capture universal characters of a cell type. Analysis of expression levels cannot be accurately annotated for cells because differences in transcription do not necessarily explain biological characteristics in terms of cellular functions and because the data themselves do not inform about the correct mapping between cell types and genes. Hence, in this study, we developed a new representation of cellular identities that can be compared over different datasets while preserving nontrivial biological semantics. To generalize the notion of cell types, we developed a new framework to manage cellular identities in terms of set theory. We provided further insights into cells by installing mathematical descriptions of cell biology. We also performed experiments that could correspond to practical applications in annotations of RNA-seq data.

INTRODUCTION

In multicellular organisms, cells (including stem cells) diverge into different cell types during development and acquire unique functions (Pasquini et al., 2021). As a result of the process, triggered by intrinsic and extrinsic stimulations by spatially specific molecules, cell fate and function are fixed to show morphological and molecular regularities (Pasquini et al., 2021; Tanay and Sebé-Pedrós, 2021). These preserved characteristics of cells have inspired analogies to Linnaean species classification, a hierarchical classification of species. Therefore, the concept of cell types is understood as hierarchically structured sets with unique morphological and functional properties of cells in a multicellular organism (Tanay and Sebé-Pedrós, 2021).

Although the emergence of single-cell RNA sequencing (scRNA-seq) has radically broadened our understanding of the heterogeneity and dynamics of cells (de Vargas Roditi and Claassen, 2015), the classic taxonomies of cell types are nevertheless essential because cell type annotation is an inevitable step toward understanding biological phenomena (Pasquini et al., 2021).

We observed that cell type annotation in scRNA-seq data can be problematic because the transcriptional differences may not always explain all biological characters in terms of morphologies and functions (Pasquini et al., 2021). Because cells undergo a smooth transition of transcriptional states rather than a clear distinction between cells (Street et al., 2018), discussion of cellular identities that adheres exclusively to transcriptional differences may not reach convincing consequences for identical cells in different phases of pseudo-time (RNA-seq data are not time series data). Cells are annotated with specific cell types based on relative differences within a dataset. Hence, it is unclear whether annotated cells can fulfill globally recognized aspects of the cell types. Although this pitfall is avoidable when we have a dataset that is large enough to represent canonical characteristics of a cell type and cells can be compared in different datasets, direct comparison is not suitable when the experimental protocols (such as sample treatment, sequencing equipment, algorithms, and methods of regularization) are inconsistent. Considering that researchers often integrate datasets obtained by others, we require an acceptable solution to this problem for the furtherance of stem cell biology and other related fields.

In this study, we developed a new evaluation metric for the similarity of cellular identities based on the structures of specific gene regulatory networks (GRNs). This metric allows comparisons of arbitrary clusters of cells in arbitrary datasets even when the population configurations of the samples are drastically different.

To reinforce our idea, we introduced a new logic of treating a group of cells in mathematical terms by defining the term "comparison of cellular identities." We also established a new method of annotation that supports our theory by introducing nontrivial background information on cellular taxonomies.

RESULTS

Definition of the generalized expression network and GRNs in this research

Although we can observe the biological functions of cells in countless aspects, we can only obtain the expression

This is an open access article under the CC BY license (http://creativecommons.org/licenses/by/4.0/).

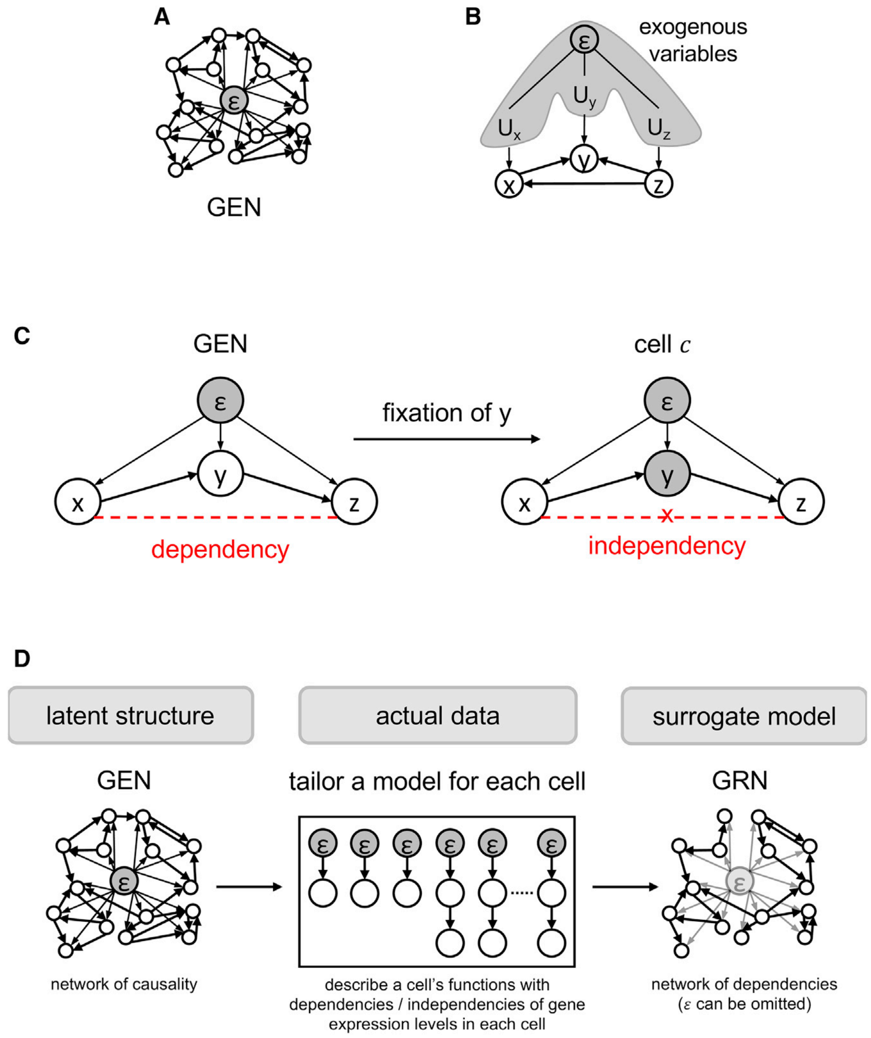

Figure 1. The GEN as a universal model of causality for gene expression and the possessed cascades as observed relationships of the genes

(A) Schematic of the GEN. The vertexes denote genes, and the edges denote causality. A latent variable ε for extrinsic factors is present in the center of the illustration.

(B) ε as the Cartesian product of the exogenous variables for the corresponding vertex in the causal graphical model.

(C) Causality blockade altering the relationship between two variables. When the variable y is unobserved, the path between x and z remains (i.e., x and z are mutually dependent on each other). In contrast, when the observation fixes the y value, the path between x and z is blocked (i.e., the two variables are mutually independent).

(D) Graphical explanation of the GEN and a GRN.

levels of genes in fixed timings from the RNA-seq data, and the entire picture of cellular dynamics is hidden. When a significant number of cells is present, we can assume cellular functions from the data by inference of the GRNs because regulatory interactions of genes control cellular functions, and GRNs are a typical representation of such interactions (Aalto et al., 2020). The structures of GRNs can be compared among different datasets even when they are processed differently. Therefore, in this study, we consider the status of dependencies and independencies of the genes as one dimension of cellular functions.

To study the transcriptome of cells, we first assumed a causal model that could fully describe the functions observable in the RNA-seq data in all cells and defined it as the original form of the GRN. To distinguish this GRN from other GRNs that are inferred from the data, we consider it the generalized expression network (GEN), which consists of the genes in G, a complete set of the genes in the RNA-seq data. Cells are influenced by extrinsic signals (such as morphogens, hormones, mechanical stresses, and cytokines.); hence, we assumed that a latent variable for the extracellular environment (defined as ε) exists as the parent of all genes in the causal graph of the GEN (ε can be interpreted as a Cartesian product of the exogenous variables U_g, where U_g corresponds to $g \in G$; Figures 1A and 1B)). In this case, the GEN is a directed multigraph (it leaves room for the feedback loops in the model), where the vertexes are the elements in $G \cup \{\varepsilon\}$, and the edges reflect causality. Exogenous variables of a causal model are defined as observed or unobserved background factors that remain unexplained; they influence, but are not influenced by, the other endogenous variables in the model (Pearl, 2009).

When quantifying RNAs in samples to obtain the scRNA-seq data, it is possible that protocols of sample collection

have some effects on the transcriptome. In such cases, some parameters in the GEN are fixed in a certain range, and the dependency and independency of the genes are affected. For example, supposing the pathway shown in Figure 1C (g_x, g_y, g_z : genes) has no dose-dependent effects, but the existence of mRNAs of those genes only controls the phenotype of cells (if g_x is expressed, then g_y is expressed, and if so, then g_z is expressed), and g_y-positive cells are collected via flow cytometry ahead of RNA-seq. In such a case, samples are limited to g_x-positive and g_y-positive cells, and g_z is no longer dependent on g_x because g_x and g_y do not have dose-dependent effects on g_z. Because the blockade of pathways in the GEN reflects cellular characteristics, we decided to consider the individual status of the cellular functions (in terms of dependency or independency of the genes) separately from the GEN (latent and universal explanations for gene interactions) and GRN because a network of gene statistical dependencies represents cellular identities (Figure 1D).

Therefore, in this study, the GEN is a universal model that considers the causality of genes, and the GRN is a descriptive model that considers the gene dependencies and independencies that can be observed in the RNA-seq data of individual cells (ε and the edges between ε and genes can be omitted because their existence is not visible in data but is evident from the definition of ε).

Biological and computational requirements for implementation

When annotating the scRNA-seq samples, three options are available. The first approach relies on a public database of cell type-specific markers and their ontologies for a biological explanation. The second method uses labeled scRNA-seq data as a reference for correlation-based scoring of the query data. The final method uses supervised learning models that involve classification tasks of cell types (Pasquini et al., 2021). These strategies have in common the belief that only a limited number of genes are required to identify cell types. Reduction of genes is advantageous for biological and computational reasons because it helps with interpretability of the results and decreases computational costs. The proposed model offers similar benefits. Because GRNs consider pairwise relations of genes, similar advantages are received with a smaller number of genes.

In general, it is untrivial that the reduced number of genes explains all of the cellular functions required in defining the cell type in Linnaean taxonomy. To check whether cellular functions (structures of GRNs) of cell type-specific genes are preserved, we mathematically describe the operation of creating the GRN of a sample ($\forall c \in S$, where S is the complete set of the samples) from the genes of interest ($\forall F \subset G$, where G is the complete set of genes), and check whether the structures of the GRNs on F are preserved between the GRNs on G and F (Figure 2A).

Before proceeding with discussion on GRNs, we define a cascade as a pair of genes denoted as $\{g_x, g_y\}_{g_x}$, where $\forall g_x, g_y \in G$ and g_x are dependent on g_y ($\{g_x, g_y\}_{g_x} = \{g_y, g_x\}_{g_x}$ and $\{g_x, g_y\}_{g_x} \neq \{g_x, g_y\}_{g_y}$). We define a complete set of cascades on G (denoted as $C(G) := \{\{g_x, g_y\}_{g_x} | g_x, g_y \in G\}$) and a set of possessed cascades of c as a subset of $C(G)$, which is a set of observed cascades in c (denoted as $C_c(G)$). When we define $f_c : G \times G \rightarrow C(G)$, which is in one-to-one correspondence with $\forall c \in S$, as shown below, $C_c(G) = f_c(G \times G)$:

$$\forall c \in S,\ \exists! f_c : G \times G \rightarrow C(G)\ s.t.\ \forall g_x, g_y \in G$$

$$f_c(g_x, g_y) = \begin{cases} \{g_x, g_y\}_{g_x} & (\text{if } g_x \text{ is dependent of } g_y \text{ in } c) \\ \{g_x\}_{g_x} & (\text{if } g_x \text{ is independent of } g_y \text{ in } c \vee g_x = g_y) \end{cases}$$

To compose a GRN from the possessed cascades of c, we consider G (which is isomorphic to $V_c(G)$ as a set) as the vertex set and define rules to draw a directed edge from g_y to g_x if $\{g_x, g_y\}_{g_x} \in E_c(G)$, where the definition of $V_c(G)$ and $E_c(G)$ are given below. It must be noted that $V_c(G) \sqcup E_c(G) \cong C_c(G)$ for $\forall l \in C_c(G)\ s.t.\ 1 \leq |l| \leq 2$, where $|l|$ is the cardinality of the set l (Figure 2B):

$$V_c(G) := \{l \in C_c(G) \,|\, |l| = 1\}$$

$$E_c(G) := \{l \in C_c(G) \,|\, |l| = 2\}$$

If the modeler prefers explicitly showing ε in the GRN, $G \cup \{\varepsilon\}$ is considered for the vertex set, and $E_c(G) \cup \{\{g_x, \varepsilon\}_{g_x} | g_x \in G\}$ is suitable for the edge set.

When we map $F \times F$, which is a subset of $G \times G$, by f_c, we obtain $C_c(F) = f_c(F \times F) \subset f_c(G \times G) = C_c(G)$, and we can compose the GRN on F under the same rule by defining $V_c(F)$ and $E_c(F)$ as follows:

$$V_c(F) := \{l \in C_c(F) \,|\, |l| = 1\} \cong F$$

$$E_c(F) := \{l \in C_c(F) \,|\, |l| = 2\}$$

If $V_c(F) \subset V_c(G)$, $E_c(F) \subset E_c(G)$, and $C_c(F) = \{f_c(g_x, g_y) | \forall g_x, g_y \in F\}$ (by its definition), then the GRN on F is an induced subgraph by F of the GRN on G (Figure 2B). The same can be applied when ε is included in the GRNs, simply by considering $F \cup \{\varepsilon\}$ as the vertex set and $E_c(F) \cup \{\{g_x, \varepsilon\}_{g_x} | g_x \in F\}$ as the edge set, and the GRN on F is the induced subgraph by $F \cup \{\varepsilon\}$ of the GRN on G with ε. Therefore, when the modeler focuses on a certain subset of genes, such as cell type-specific genes, its function (edges in the GRN) is preserved. In other words, the structures of GRNs are not changed by ignoring

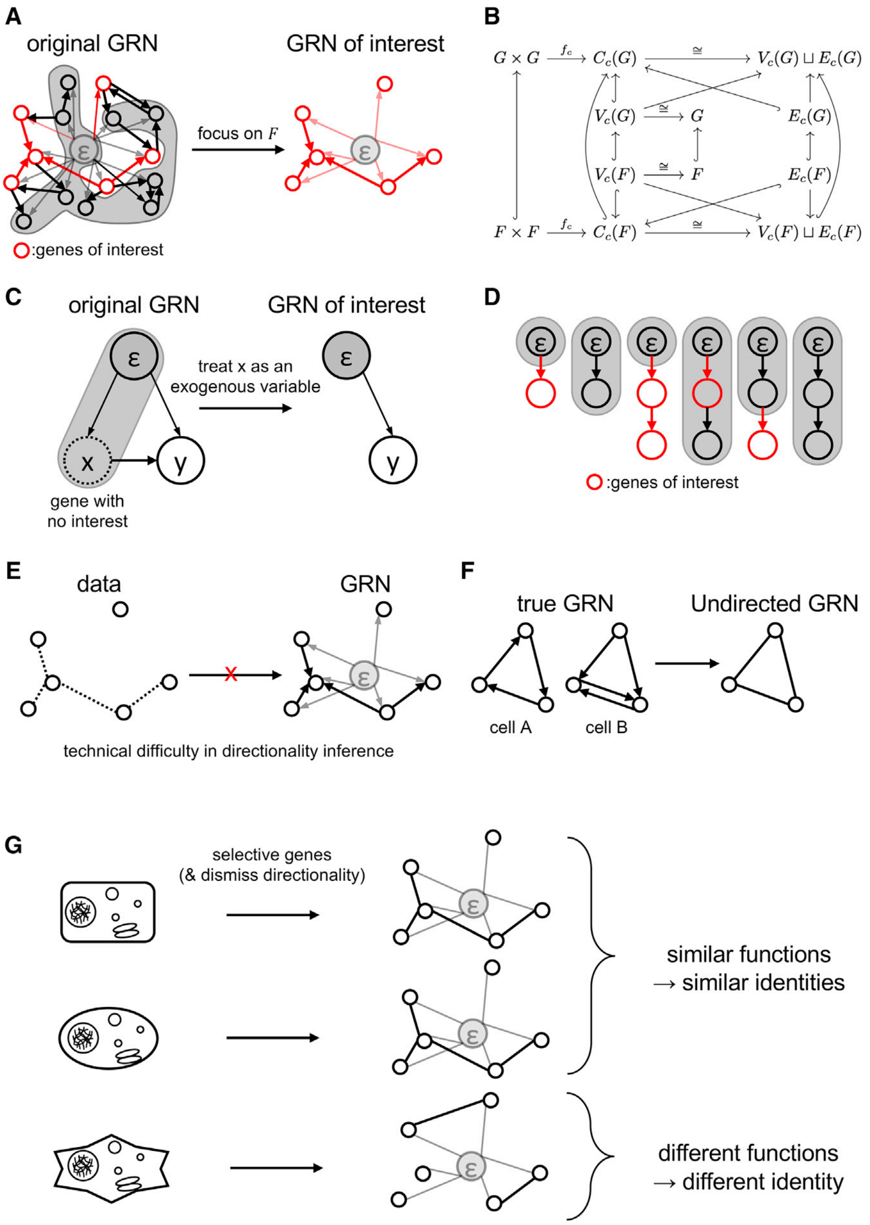

Figure 2. Mathematical operations required to simplify the GRN

(A) A conceptual illustration of excluding genes from the GRN.

(B) Graphs of the genes of interest are induced subgraphs of the graph featuring all genes.

(C) An operation to remove genes from GRNs, which is equivalent to treating the genes as exogenous variables.

(D) Cascades and vertexes treated as exogenous variables. Shaded areas are neglected in the simplified GRN.

(E) Technical difficulties of data-driven causal exploration.

(F) Effect of conversion from directed to undirected edges.

(G) Comparison of cellular identities using undirected GRNs.

all of the irrelevant genes. Hence, the modeler can remove thousands of genes from the GRNs without losing any information about the cellular functions of the genes of interest.

Although this explanation reveals the effectiveness of GRNs for selective genes, it is still unclear how the ignored genes ($\forall h \in H : = G - F$) are to be treated. If the ignored genes can be treated as exogenous factors, then they can be treated as ε, which is a nontrivial conclusion. To elucidate this point, we introduced an algebraic structure called tropical semiring (Grigg and Manwaring, 2007) to each cascade and defined mathematical operations to treat certain genes as equivalent to ε by considering ideals on the semiring (Figure 2C).

Tropical semirings are algebraic structures in tropical geometry that are applied in theoretical computer science among other fields. They have been named in honor of the Brazilian computer scientist Imre Simon (Katz, 2017; Pin, 2010; Simon, 1978). Further information on tropical semirings is provided in the Supplemental experimental procedures (Katz, 2017; Mitchell and Sinutoke, 1982).

In this study, into $l_g \cup \{\varepsilon\}$ (where $\forall l_g \in C(G)$, $\exists ! g \in l_g$, $\forall g_x \in l_g$ *s.t.* $g = g_x \vee g$ *is dependent on* g_x), we introduced two binary operations, addition (denoted as $\oplus_g$) and

multiplication (denoted as $\odot_g$), so that the tropical semiring is defined as follows:

$$\forall \tilde{g}_x,\ \tilde{g}_y \in l_g \cup \{\varepsilon\}$$

$$\tilde{g}_x \oplus_g \tilde{g}_y = \begin{cases} \tilde{g}_x \left(if\ \tilde{g}_x = g \vee \tilde{g}_y = \varepsilon \vee \tilde{g}_x = \tilde{g}_y \right) \\ \tilde{g}_y \left(if\ \tilde{g}_y = g \vee \tilde{g}_x = \varepsilon \vee \tilde{g}_x = \tilde{g}_y \right) \end{cases}$$

$$\tilde{g}_x \odot_g \tilde{g}_y = \begin{cases} \tilde{g}_x \left(if\ \tilde{g}_y = g \right) \\ \tilde{g}_y\ (if\ \tilde{g}_x = g) \\ \varepsilon\ (else) \end{cases}$$

$\forall l_g \in C(G)$, $(l_g \cup \{\varepsilon\},\ \oplus_g,\ \odot_g)$ is a commutative semiring, and $z_g : l_g \cup \{\varepsilon\} \rightarrow \{0,\ 1,\ \infty\}$, defined below, is a semiring homomorphism when $\forall \tilde{g}_x \in l_g \cup \{\varepsilon\}$, and $(\{0,\ 1,\ \infty\},\ \oplus,\ \odot)$ is a tropical semiring. Additional information on commutative semirings and semiring homomorphism is provided in the Supplemental experimental procedures (Pin, 2010; Zumbrägel and Zelmanov, 2008).

$$z_g : \tilde{g}_x \mapsto \begin{cases} 0\ (if\ \tilde{g}_x = g) \\ \infty\ (\ if\ \tilde{g}_x = \varepsilon) \\ 1\ (else) \end{cases}$$

Next, we introduced ideals of the semirings to describe mathematical operations to consider $\forall h \in H$ and ε as exogenous factors (i.e., ε) without losing information related to $\forall g_x \in F$ by utilizing properties of quotient semirings and the natural homomorphism. Additional information on quotient semirings, natural homomorphism, and ideals of a semiring are provided in the Supplemental experimental procedures (Allen, 1969; Zumbrägel and Zelmanov, 2008).

Prior to the construction of ideals, we define a map that shows one-to-one correspondence to $\forall l \in C(G)$ as follows:

$$\forall l \in C(G),\ \exists ! i_l : G \cup \{\varepsilon\} \rightarrow l \cup \{\varepsilon\}\ s.t.$$

$$\forall \tilde{g}_x \in G \cup \{\varepsilon\},\ i_l\ : \tilde{g}_x \mapsto \begin{cases} \tilde{g}_x (\ if\ \tilde{g}_x \in l) \\ \varepsilon\ (else) \end{cases}$$

Using this map, we define an ideal I_{l_g} on a semiring $(l_g,\ \oplus_g,\ \odot_g)$ as follows:

$$I_{l_g} :\ = \left\{ \sum_{\tilde{h} \in H \cup \{\varepsilon\}} g_x \odot_g i_{l_g}(\tilde{h}) | g_x \in l_g \right\},$$

where $\forall l_g \in C(G),\ \exists ! i_{l_g} : G \cup \{\varepsilon\} \rightarrow l_g \cup \{\varepsilon\}$ and $\forall \tilde{h} \in H \cup \{\varepsilon\}$. Using the definition of I_{l_g}, a natural homomorphism $\pi_{l_g} : l_g \cup \{\varepsilon\} \rightarrow l(g \cup \{\varepsilon\}) / I_{l_g}$ can be defined. For a better understanding of π_{l_g}, the fiber of $[\varepsilon]$, $\pi_{l_g}^{-1}([\varepsilon]) = \{\tilde{g}_x \in l_g \cup \{\varepsilon\} | \pi_{l_g}(\tilde{g}_x) = [\varepsilon]\}$, is defined as follows:

$$\pi_{l_g}^{-1}([\varepsilon]) = \begin{cases} \{\tilde{g}_x \in l_g \cup \{\varepsilon\} | \forall \tilde{g}_x \notin F\}\ (if\ \tilde{g} \in F) \\ l_g \cup \{\varepsilon\}\ (if\ \tilde{g} \in H) \end{cases}$$

where $\exists ! \tilde{g} \in l_g \cup \{\{\varepsilon\},\ \tilde{g} \neq \varepsilon$ is the neutral element of multiplication of the tropical semiring $(l_g \cup \{\varepsilon\},\ \oplus_g,\ \odot_g)$. A conceptual diagram of the image of $\pi_{l_g}^{-1}$ is shown in Figure 2D. Given that, we defined $\overline{C_c}(G)$, $\overline{V_c}(G)$, and $\overline{E_c}(G)$ as follows:

$$\overline{C_c}(G) :\ = \left\{ l_g \cup \{\varepsilon\} - \pi_{l_g}^{-1}([\varepsilon]) | \forall l_g \in C_c(G),\ \exists ! \pi_{l_g} : l_g \cup \{\varepsilon\} \rightarrow \left(l_g \cup \{\varepsilon\} \right) / I_{l_g} \right\}$$

$$\overline{V_c}(G) :\ = \left\{ \overline{l_g} \in \overline{C_c}(G) \middle| \left| \overline{l_g} \right| = 1 \right\} = V_c(F) \cong F$$

$$\overline{E_c}(G) :\ = \left\{ \overline{l_g} \in \overline{C_c}(G) \middle| \left| \overline{l_g} \right| = 2 \right\} = E_c(F)$$

Drawing a directed graph according to the rules above, the graph coincides with the GRN on F. $\overline{C_c}(G) - \{\varnothing\} = C_c\{F\}$, and the definitions of $\overline{V_c}(G)$ and $\overline{E_c}(G)$ correspond to those of $V_c(G)$ and $E_c(G)$, respectively. As the series of mathematical operations gives rise to the GRN on F, it is proven that the ignored genes are treated equivalent to exogenous factors.

So far, we discussed the effect of gene elimination from GRNs on the GRN structures. Originally, this topic was required in the interest of biological and computational limitations. Regarding limitations of GRN implementation, directionality of the graph is an issue. Considering a gene g_x and its regulatory gene g_y (e.g., a transcription factor) in the GEN, a directed edge must exist from g_y toward g_x because there is a causality between them, and there is no edge in the opposite direction unless the feedback loop exists. In contrast, it is fairly possible that an edge from g_y to g_x and one in the opposite direction exist in the GRN because an edge in the GRN represents statistical dependency. In such a case, the opposite edge has no biological relevance. Hence, undirected edges can be more interpretable rather than directed edges in some cases. There is a technical benefit in converting directed into undirected edges. To investigate statistical dependencies, the Peter and Clark (PC) algorithm of Bayesian network structural learning (Spirtes et al., 2000) is a suitable option for creating GRNs. However, a Bayesian network is a directed acyclic graph (DAG) with a conditional probability distribution in each edge (Cheng et al., 1997), whereas a GRN is not confirmed to be a DAG; therefore, an ad hoc process is required to dismiss the directionality of edges (Figure 2E). Because the process is merely equivalent to application of equivalence relations $\{g_x,\ g_y\}_{g_x} \sim \{g_x,\ g_y\}_{g_y}$ to $C_c(G)$, the existence or absence of the edges between two arbitrary

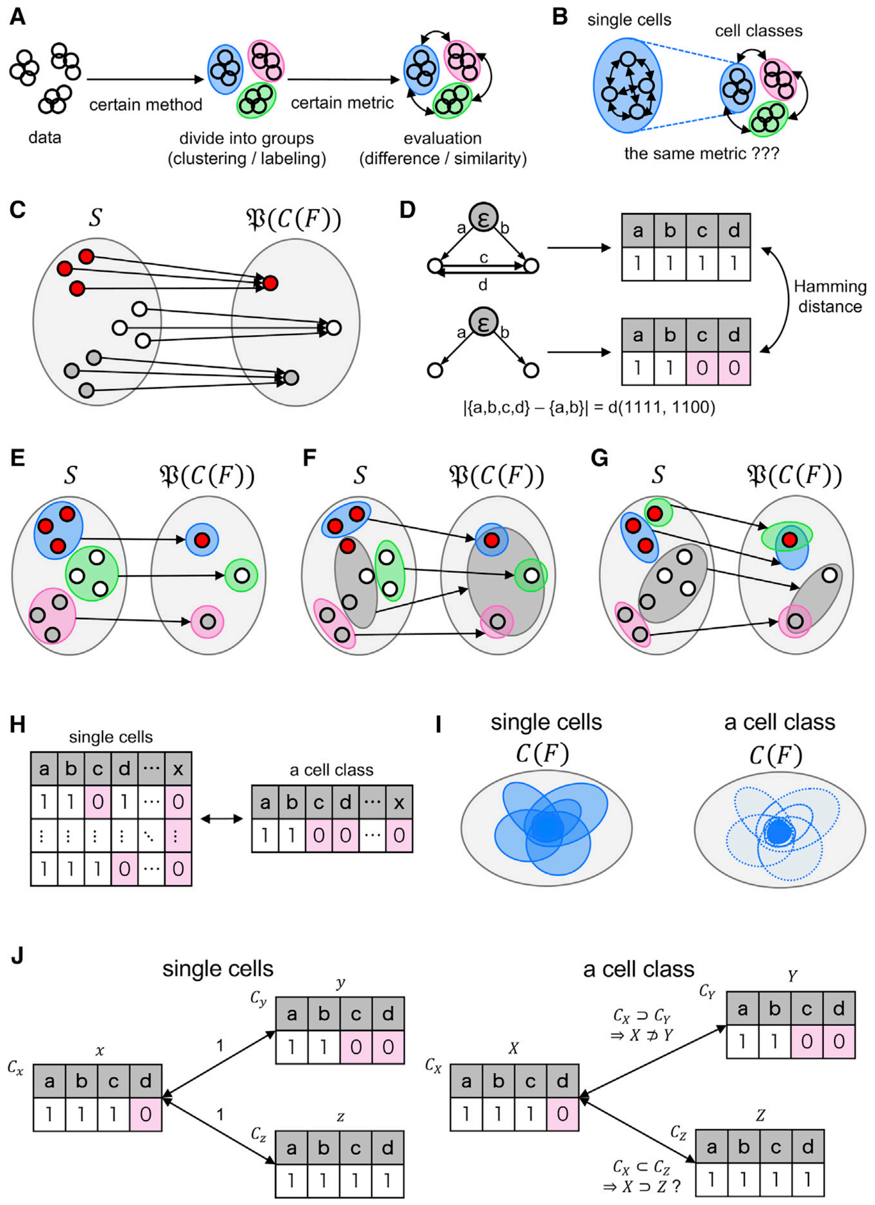

Figure 3. Topological spaces of cellular similarity

(A) Standard workflow of scRNA-seq analysis.

(B) Using the same metric to compare single cells and cell classes, which is nontrivial.

(C) Morphism that maps single cells to the corresponding GRNs.

(D) Structural similarity of single cells' GRNs, which is equivalent to the Hamming distance for strings of 0 and 1.

(E–G) Quotient spaces of cell classes. The shaded areas in S are cell classes, and those in $\mathfrak{P}(C(F))$ are images of φ pertaining to the cell classes.

(H) Graphical explanation of the statement regarding the choice of representative GRNs from a cell class.

(I) The GRN of a cell class, approximately regarded as the intersection GRN for all single cells in the cell class.

(J) The inclusion relations of eigen-cascades, indicating the structural similarity of GRNs in the case of cell classes.

genes is stably preserved through the process. Undirected GRNs lose resolution to distinguish between the different cells when there is no other way to discern between them other than the directionality of the edges (Figure 2F).

Consequently, we checked the biologically and computationally preferred operations for creating GRNs, subsequently establishing a basis to compare cellular identities with the GRNs of cells (Figure 2G).

Cell classes in scRNA-seq data: Data-driven definitions of cells' identities

In Figure 3A, an example of a workflow in RNA-seq data analysis is illustrated. First, samples are classified using certain methods, followed by elucidation on the groups. The choice of method matters because we prefer to pay attention to the biological and computational aspects in clustering (Kiselev et al., 2019). In some cases, we stratify them with supervised labels (e.g., information on cell lines, patients, drug treatment, etc.) instead of clustering. The expected result of the clustering or stratification is a state where the groups share some properties within themselves. In other words, we classify samples into equivalent classes under a certain configuration of the equivalent relation. The equivalent classes have an explicit reasoning for classification in data including labels; therefore, we call them cell classes to distinguish them from empirical cell types. A cell

class can be named after a cell type (e.g., neurons), but this does not imply that the cell class is a necessary and sufficient set to represent the cell type. Hence, we need a new concept for cells of an identity.

Subsequently, the cell classes are compared in certain metrics. Because it is not self-evident that diversity in morphologies and functions can be seen as distances of the scRNA-seq data (Pasquini et al., 2021), in this study we aim to develop a new metric based on the dependencies of genes, the GRN. A good method of evaluating cell similarities has not yet been developed. The optimal metrics for comparing single cells or cell classes may differ (Figure 3B). Therefore, we established an evaluation metric for cell classes.

First, the notations to be used in the following discussion are set. If we describe the process of creating a GRN (regarding $\forall F \subset G$) of a cell as a morphism, $\varphi : S \rightarrow \mathfrak{P}(C(F))$ is a suitable definition, where $\mathfrak{P}(C(F))$ is the power set of $C(F)$, for $\forall c \in S$, $C_c(F) \subset C(F)$ (Figure 3C), provided, similarity of cell c and c' can be interpreted as similarity of $\varphi(c)$ and $\varphi(c')$. We define a morphism to classify samples to cell classes, where $\forall c,\ c' \in S,\ c \sim c' :\Leftrightarrow c$ and c' belong to the same cell class, as follows:

$$\forall \sim : \textit{equivalence relation},\ \exists ! \xi : S \rightarrow S/\sim\ s.t.\ \forall c \in S,\ \xi(c) = [c]$$

It must be noted that $\sim$ can be configured arbitrarily and defined independent of φ because the cell classes can be defined arbitrarily and separately from the GRN structures.

Subsequently, we define a metric to compare the identities of the single cells based on the GRN structures and attempt to expand it to cell classes. When comparing the single cell identities by the GRN structures, we match the edges in the GRNs (the operation can be described as $d(c, c') = |\varphi(c) \cup \varphi(c')| - |\varphi(c) \cap \varphi(c')|$, where $d : S \times S \rightarrow \mathbb{R}$ is the pseudo-metric (not a distance function because $d(c, c')$ can be 0 even when $c \neq c'$), and $\forall c,\ c' \in S$; $\mathbb{R}$ is the set of real numbers. Therefore, it is equivalent to the comparison of character strings where the positions in the strings correspond to the index of edges, and the numbers represent the existence or absence of edges. Hence, we define the evaluation metric of single cells based on the GRN structures as the Hamming distance of the GRN structures (Figure 3D). Additional information on Hamming distance is provided in the Supplemental experimental procedures (Bookstein et al., 2002).

The topological space $(S, \mathscr{O})$ can be defined using the open ball $B_\epsilon(c)$ as follows:

$$B_\epsilon(c) : = \{c' \in S | \exists \epsilon \in \mathbb{R}_{>0}\ s.t.\ d(c, c') < \epsilon\}$$

$$\mathscr{O} : = \{o \subset S | \forall c \in o,\ \exists \epsilon \in \mathbb{R}_{>0}\ s.t.\ B_\epsilon(c) \subset o\},$$

where $\forall c \in S$, d is the Hamming distance function, and $\mathbb{R}_{>0}$ is the set of positive real numbers. $(S, \mathscr{O})$ is a pseudo-metric space. Additional information on the topological space, open ball, and pseudo-metric space is provided in the Supplemental experimental procedures (Herrlich and Keremedis, 2015; Manetti, 2015).

Subsequently, we present a discussion on cell classes by considering images of cell classes under φ in variable cases. Supposing that, when the definition of cell classes coincides with the GRN (that is, $\forall c \in S,\ \xi(c) = \{c' \in S | \varphi(c') = \varphi(c)\}$; Figure 3E), $\varphi(\xi(c)) = \{\varphi(c)\}$. Hence, when we define $\overline{\mathscr{O}} : = \{\overline{o} \subset \xi(S) | \xi^{-1}(\overline{o}) \in \mathscr{O}\}$, where $\xi^{-1}(\overline{o})$ is the fiber of $\overline{o}$, $(\xi(S), \overline{\mathscr{O}})$ is defined as a metric space because the metric reflection (the equivalence relation $c \sim c' :\Leftrightarrow d(c, c') = 0$) has been introduced in the pseudo-metric space (Herrlich and Keremedis, 2015). Therefore, in this case, the Hamming distance is suitable for cell classes as well.

We consider the second case (Figure 3F), where $\exists ! [c] \in \xi(S),\ \forall [c'] \in \xi(S),\ [c] \neq [c']\ s.t.\ \forall c'_1,\ c'_2 \in [c'],\ \exists c_1, c_2 \in [c],\ \varphi(c'_1) = \varphi(c'_2) = \varphi(c_1) \neq \varphi(c_2)$ (one cell class covers all patterns of the GRNs, and the other cell classes have only one and unique GRN structures). In this case, the quotient space $(\xi(S), \overline{\mathscr{O}})$ is an indiscrete space because $\overline{\mathscr{O}} : = \{\overline{o} \subset \xi(S) | \xi^{-1}(\overline{o}) \in \mathscr{O}\} = \{\varnothing,\ \xi(S)\}$. Hence, the Hamming distance is not suitable for the cell classes in this case.

Although the second case may be an impractical proposition, the Hamming distance is not always suitable for evaluation of cell classes because, in certain cases, the GRN structures within a cell class are not uniform, and the situation makes it impossible to define the Hamming distance of the GRNs (Figure 3G). To resolve this problem, we propose a hypothesis, as described below (Figure 3H), that is also preferred in terms of implementation because the statements capture the properties of statistical tests of independence in such a way that the GRNs can be surmised by checking the dependencies and independencies of genes from a collective expression matrix of a cell class, not single cells.

1. If a certain proportion of cells in the cell class do not have an edge with respect to specific genes, then the edge does not exist in the GRN of the cell class.
2. If an edge exists in the GRN of a cell class, then it also exists in the GRNs of a major proportion of cells belonging to the cell class.

The vague descriptions of "certain proportion" and "a major proportion" are used to adopt uncertainty of the statistical tests of independence. We adopt Pgmpy, a Python package for Bayesian networks (Ankan and Panda, 2015), where the chi-square test is used for the categorical variables, and hypothesis testing for the Pearson correlation coefficient is used for the continuous variables; both models hypothesize the independence (or no correlation) of variables as the null

hypothesis. The first statement considers a situation where roughly evenly sized populations with and without edges are concatenated as one cell class, and the null hypothesis may hold for the cell class, although the alternative hypothesis holds in the subpopulation with the edge. In contrast, the second statement claims that there must be a sufficient number of cells with the edge in the cell class to defy the null hypothesis. Because the concrete threshold to fulfill them differs depending on the dataset and p values, we decided to describe it as above.

Given the statements, we can define a set of cascades for a cell class, called the eigen-cascades of the cell class. We denote the eigen-cascades on F of a cell class $[c]$ as $C_{[c]}(F)$. If we set a small p value and allow minor exceptions by considering them as noise, then we can regard $C_{[c]}(F)$ as $\bigcap_{c\in[c]} C_c(F)$ (Figure 3I).

Next, we present a discussion on the suitable metric for similarities of the cell classes. Although we have eigen-cascades, we cannot directly apply the Hamming distance because some eigen-cascades can include other eigen-cascades as a subset (Figure 3J). When we consider the eigen-cascades of a cell class as a set of required aspects of the cell class, a cell class $[c]$ is a subset of a cell class $[c']$ when the eigen-cascades of $[c]$ are a superset of the eigen-cascades of $[c']$, in the same way as a ring is a semiring, but the opposite is false. Therefore, we need an asymmetric evaluation function that expresses this asymmetric inclusion relation between $[c]$ and $[c']$.

Here we propose an evaluation metric for cell classes $d^* : \xi(S)\times\xi(S)\rightarrow\mathbb{R}$ as follows:

$$d^*(\xi(c),\ \xi(c')) :\ = 1 - \frac{|\varphi^*(\xi(c))\cap\varphi^*(\xi(c'))|}{|\varphi^*(\xi(c))|} = 1 - \frac{|C_{[c]}(F)\cap C_{[c']}(F)|}{|C_{[c]}(F)|},$$

where $\forall c,\ c'\in S$, and $\varphi^* : \xi(S)\rightarrow\mathfrak{P}(C(F))$ is a morphism that maps cell classes to the corresponding eigen-cascades. It is a quasi-pseudo-metric that is not continuous from the pseudo-metric space; that is, the topological space of the single cells. Additional information on quasi-pseudo-metrics is provided in the Supplemental experimental procedures (Kũnzi, 1992).

Regarding the biological merits of considering cell classes, arbitrary cell classes can be strictly defined with a set of cascades and can be defined flexibly by the modeler by adding the desired cascades (such as previously known cascades, cascades of interest to the authors, and ones that are found in data analysis) as the eigen-cascades of the cell class to highlight the characteristics of the cells. The concept of the GRN and cell classes can be applied in various ways. We list some examples below:

1. Inference of similarity: comparison of cell classes on eigen-cascades
2. Annotation: comparison between cell classes in referential and query data
3. Multifaceted description: definition of cell classes in multiple criteria

Implementation of cell class annotation

To demonstrate some practical examples of application of GRNs and cell classes, we implemented annotation of RNA-seq data based on the GRN structures. The process consists of three steps: formation of cell classes, feature selection for the GRNs, and comparison of the eigen-cascades (Figure 4A). To capture the canonical features, we used two datasets for the reference and query. We also performed manual annotation using a conventional approach (Figure 4B), comprising dimensionality reduction, clustering, searching differentially expressed genes (DEGs), and interpretation of clusters based on the DEGs, aiming to validate the results and compare the performance with our method.

In the annotation with the GRNs, cell classes in the referential and query data are compared with respect to their eigen-cascades; hence, both datasets must be divided into clusters using various methods. Because the model of GRN itself does not require a specific approach for defining the cell classes, any method and tool are suitable. In this study, because we preferred to reflect the Linnaean hierarchical taxonomies in the data-driven methods of annotation, we used factor analysis and k-means clustering, aiming to explain the data using latent variables that can be interpreted in domain-specific aspects. First, a query dataset is processed using factor analysis on cell type-specific markers, as described in previous studies.

Second, we selected genes to compose the GRNs. To characterize cell classes with biologically meaningful models, cell classes should be defined to represent certain cell types, and accordingly, GRNs should include characteristic genes as vertexes. Among the various methods available for feature selection, we selected marker genes that showed high communalities in the referential dataset and genes with high feature importance (FI) values in a gradient-boosting decision tree (GBDT) model with L1 and L2 regularizations to predict cell types to salvage the aspects missing from the previously reported markers. We used cell type information attached to the referential data for the ground truth of the machine learning (ML) model. We adjusted the class imbalance by random under-sampling to treat all cell types as equally important (Figure 4C).

The final step is to compare the GRNs of the referential and query data. To calculate the GRN structure, we used the PC algorithm and converted the GRNs into undirected graphs. For the evaluation metric of the comparison analysis, we applied d^*. $\forall[c]\in\xi(S)$, $|C_{[c]}(F)| = |V_{[c]}(F)| + |E_{[c]}(F)|$, where $V_{[c]}(F)$ and $E_{[c]}(F)$ are defined as

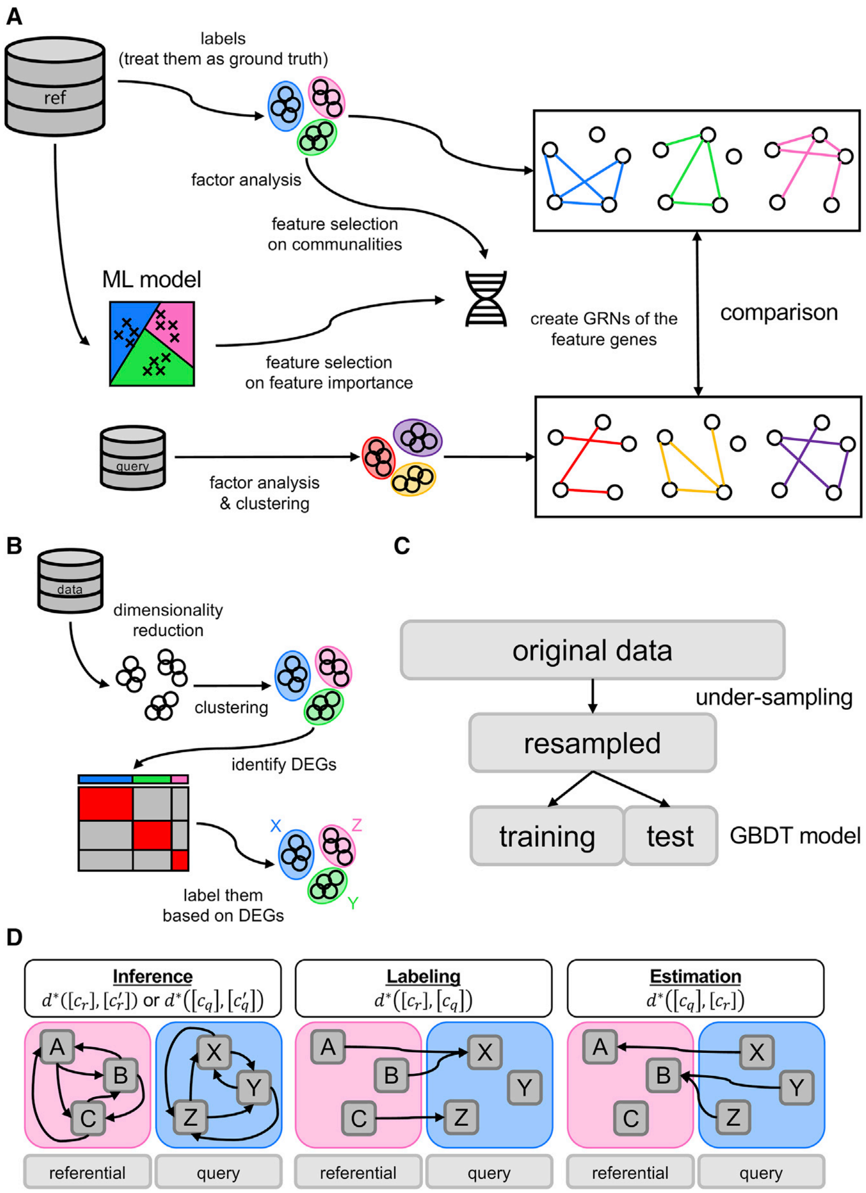

Figure 4. Overview of analyses used in this research

(A) Workflow of GRN-based cell-class annotation, using FA and an ML model.
(B) Standard workflow of the conventional annotation using DEGs.
(C) Overview of data splitting.
(D) Schematics of the three GRN-based annotation methods: inference, labeling, and estimation.

follows to indicate the vertexes and edges excluding ε and the corresponding edges:

$$V_{[c]}(F) : = \{ l \in C_{[c]}(F) || l| = 1 \}$$

$$E_{[c]}(F) : = \{ l \in C_{[c]}(F) || l| = 2 \}$$

In the application of GRNs annotation, we decided to relate pairs of cell classes in the referential and query data that minimized the d^* values. Considering d^* is an asymmetric function, when S_r is the referential data, S_q is the query data, $\forall c_r, c'_r \in S_r$ and $\forall c_q, c'_q \in S_q$ are cells, and $[c_r], [c'_r] \subset S_r$ plus $[c_q], [c'_q] \subset S_q$ are cell classes, comparisons of cell classes in the reference and query data can be performed in multiple ways, which are described in the following section (Figure 4D).

1. $d^*([c_r], [c'_r])$ or $d^*([c_q], [c'_q])$: similarities of cell classes within the same dataset (the operation is named "inference")
2. $d^*([c_r], [c_q])$: similarities from the perspective of cell classes in the referential data (the operation is named "labeling")
3. $d^*([c_q], [c_r])$: similarities from the perspective of cell classes in the query data (the operation is named "estimation")

In this study, we performed labeling and estimation.

Annotations in human brain samples

As the first demonstration of actual implementation, we conducted annotation in the scRNA-seq data of normal cells of the central nervous system (CNS), assuming a situation where we desired to annotate excitatory and inhibitory neurons in the scRNA-seq data from human fetal brain. For the referential data, a dataset named Human M1 10x (denoted m1_10x for short) from the Allen Institute for Brain Science, Seattle, Washington (https://portal.brain-map.org/atlases-and-data/rnaseq/human-m1-10x) was selected because it contained abundant samples. For the query data, GSE165388 collected by Yu et al. (2021) (scRNA-seq data from the subpallial tissues in human fetal brain from gestational week 9 [GW9] to GW12, when interneuron neurogenesis is actively taking place) was selected. Although the authors of the original study integrated datasets from GW9–GW12 (denoted as gw9, gw10, gw11, and gw12), we processed them separately to avoid overlooking batch effect.

Manual annotation by the conventional method

First we conducted manual annotations using the conventional method. We processed gw9–gw12 to identify 2,000 variable features each. The matrices were decomposed by truncated singular value decomposition (SVD), and the dimensionality of the data was estimated by parallel analysis (Figures S1A–S1D) with randomly permutated data matrices as null models (Ledesma and Valero-Mora, 2007; Ledesma et al., 2015). After clustering was performed using the shared nearest neighbor (SNN) algorithm, and DEGs were identified by Wilcoxon rank-sum test, the clusters were annotated after the DEGs with top 10 log fold change (FC) values (Figure S1E). To cope with the arbitrariness and subjectivity in interpreting DEGs by predefining clear criteria, we collected 90 marker genes in total for glia, astrocytes, oligodendrocytes, oligodendrocyte precursor cells (OPCs), microglia, neurons, excitatory neurons, caudal ganglionic eminence (CGE), lateral ganglionic eminence (LGE), medial ganglionic eminence (MGE), neural progenitor cells (NPCs), neural stem cells (NSCs), red blood cells (RBC), endothelial cells, and epithelial cells (*SLA1A2*, *VIM*, and *AQP4* as glial markers; *GFAP*, *S100B*, *SLC1A3*, *ALDH1L1*, *BYSL*, *GJA1*, *GLUL*, and *PYGB* as astrocytic markers; *OLIG2*, *CNP*, *CA2*, *NFIA*, and *NFIB* as oligodendrocyte markers; *PDGFRA* as an OPC marker; *SPP1*, *TMEM119*, *ITGAM*, *PTPRC*, and *AIF1* as microglial markers; *RBFOX3*, *TBR1*, *SOX5*, *DCX*, *BCL11B*, *FEZF2*, *SATB2*, *CUX1*, *CUX2*, *POU3F2*, *POU3F3*, and *TUBB3* as neuronal markers; *NEUROD1*, *NEUROD2*, *NEUROD6*, and *GRIN2B* as excitatory neuron markers; *CALB2*, *RELN*, *NR2F1*, *NR2F2*, and *VIP* as CGE markers; *SIX3*, *ISL1*, and *EBF1* as LGE markers; *LHX6*, *LHX8*, *MAF*, *SST*, *ERBB4*, *SOX6*, and *NKX2-1* as MGE markers; *HES1*, *HES5*, *TYMS*, *FABP7*, *EOMES*, *NEUROG1*, *NCAM1*, *TTYH1*, *DLX2*, *GAD2*, *ASCL1*, *NEUROG2*, *PROX1*, *TOP2A*, *NUSAP1*, *NR2E1*, and *CD24* as NPC markers; *PAX6*, *NES*, *SOX1*, *SOX2*, *MCM2*, *PCNA*, *MKI67*, *FOXO3*, *BHLHE22*, and *REST* as NSC markers; *HBB*, *HBM*, *HBA1*, and *HBA2* as RBC markers; *IGFBP7* and *PECAM1* as endothelial markers; *KRAT14*, *KRAT16*, *KRT17*, and *EMX2* as epithelial markers), as reported in previous studies (Jain et al., 2022; Lier et al., 2021; Lu et al., 2019; Murthy et al., 2014; Wright-Jin and Gutmann, 2019; Yu et al., 2021; Zhang and Jiao, 2015). We discarded all genes, excluding the genes listed above (when multiple genes that represented different cellular identities were suggested as DEGs for a cluster, the gene with a higher log FC value was adopted). The expression patterns of the representative genes were also checked for confirmation (Figure S1F). Although some clusters were annotated with the same identity, the expression patterns of the DEGs differed (the top 3 genes of the highest log FC values for each cluster are shown in Figures S2A–S2D). Hence, it could not be ascertained from only these results whether we could consider clusters of a name as homogeneous.

Feature selection using GBDT

We constructed a GBDT model with L1 and L2 regularizations to predict the cellular identity from the expression matrix while the cell type information attached to m1_10x was treated as the ground truth.

Prior to constructing the ML model, we verified the accuracy of the classification on m1_10x. The samples were annotated as GABAergic, glutamatergic, or non-neuronal (Figure 5A), and the classification appeared to reflect canonical taxonomies because the expression patterns of the representative marker genes corresponded to the labels (Figure S3A). Because the aim of the ML model was to identify a minimal set of genes that could computationally behave as good features of the canonical taxonomies, we rebalanced the sample sizes in each category by random undersampling to avoid underestimating the minor classes (Figure S2B). Upon resolving class imbalance, we randomly separated the dataset into training and test data. Subsequently, to reduce computational costs, the top 1,000 genes of the highest standard deviation (SD) of the mean within a class were selected as features in the GBDT model and were confirmed to include the canonical marker genes (Figures 5C and 5D).

Subsequently, a GBDT model for multiclass classification to predict labels of m1_10x with 5-fold cross-validation for hyperparameter tuning was constructed. We used "StratifiedGroupKFold" of scikit-learn to capture the universal features of the cell types shared among the subclasses. The labels of subclasses were also attached to m1_10x

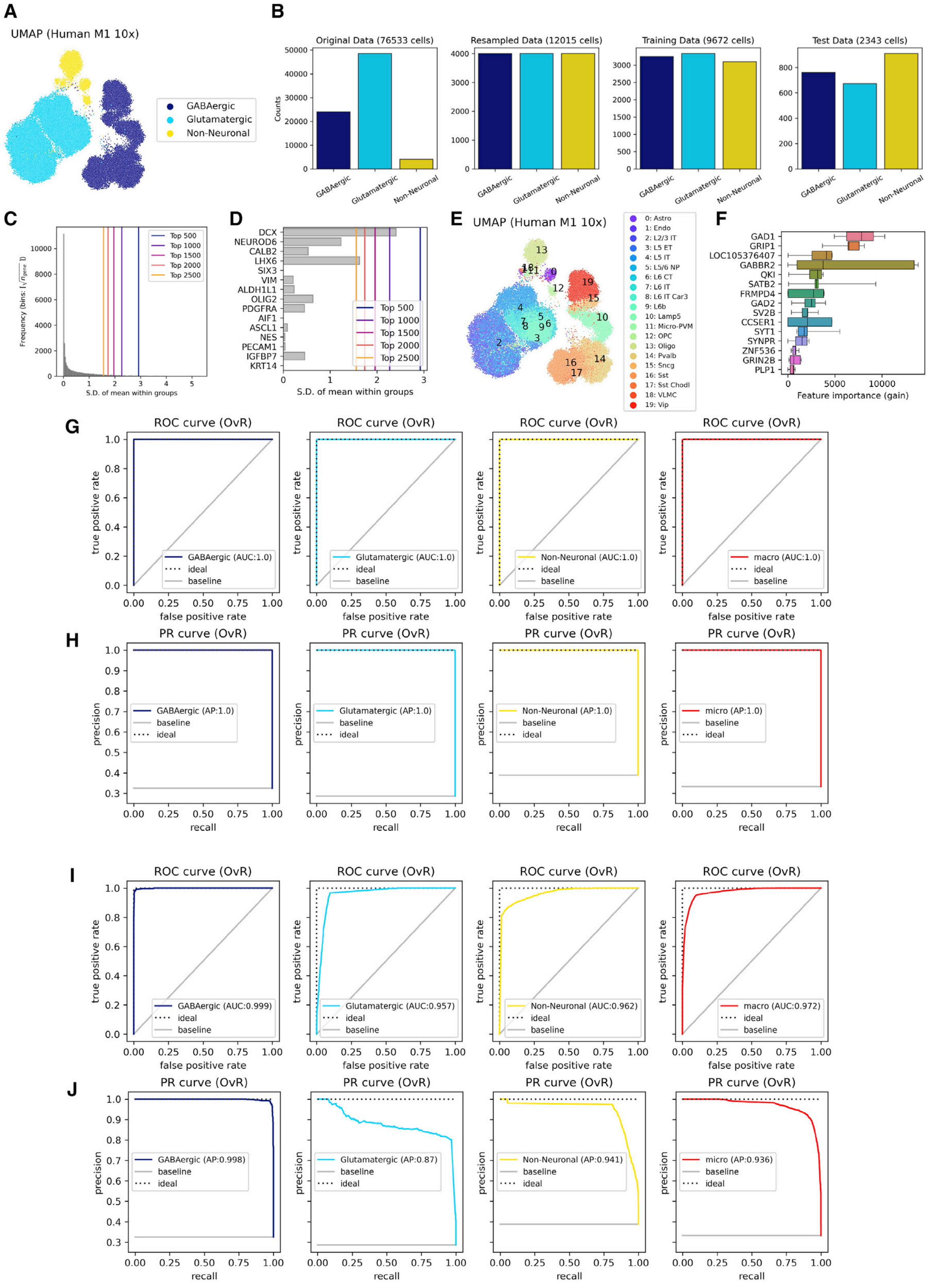

(legend on next page)

(Figure 5E). Consequently, *GAD1* and *GRIP1* were the only genes that did not score 0 in FI in any fold (Figure 5F). Because the top 15 genes with the largest FI showed high ranks of SD of the group-wise mean values, the result suggested that the feature engineering was appropriate (Figure S3B).

The performance of the GBDT model was evaluated in the one-versus-rest (OvR) classification using the area under the curve (AUC) of the receiver operating characteristic (ROC) curve and average precision (AP) of the precision-recall (PR) curve. The macro average of the AUC and the micro average of the AP were also calculated (Figures 5G and 5H). Because the GBDT model showed perfect scores, the classification function in m1_10x was considered to be very simple. When another GBDT model was constructed using only *GAD1* and *GRIP1* as the features, the new model also scored significantly high in each metric (Figures 5I and 5J). Thus, it could be assumed that the genes reflected the differences in the phenotypes of the cells to a certain degree, and it was reasonable to include them in the GRNs. *GAD1* codes glutamic acid decarboxylase, which catalyzes synthesis of gamma-aminobutyric acid (GABA) (Edelhoff et al., 1993), and *GRIP1* has been reported to correspond to learning and memory via regulation of synaptic plasticity (Tan et al., 2020).

The ML model is ineffective for automatic annotation because the category distribution is drastically distorted from that of the canonical states of CNS cells in the process of undersampling (Figure S3C).

Exploratory factor analysis on m1_10x

We performed a factor analysis of the resampled m1_10x data to identify suitable features for GRNs and verify that factor analysis is appropriate for scRNA-seq data. In exploratory factor analysis, the model should be made up of comprehensible and representative variables. Therefore, we used the 90 marker genes and calculated the Kaiser-Meyer-Olkin (KMO) criterion; the measure of sampling adequacy (MSA) was calculated for each variable (Figure S4A), and genes with an MSA of less than 0.6 were removed. Using parallel analysis, the initial number of factors was determined (Figure S4B); then, the factors with small loadings (absolute value < 0.5) were eliminated from the model. Latent factors are not guaranteed to be orthogonal; hence, we selected the quartimin rotation. In the final iterative computation results, marker genes of common cellular identities showed similar values of factor loadings (Figure S4C). Communalities and uniquenesses were calculated, and variables with high communalities were considered to reflect the effects of latent variables underlying the phenomenon (Figure S4D). The original matrix was linearly converted into a matrix with a lower dimensionality from which we could biologically interpret the bases; hence, we assumed that distances between the samples reflect the similarities of the biological characteristics. Therefore, we applied k-means clustering to the factor score matrix, and the k value that maximized the mean silhouette coefficient was considered as providing the optimal number of clusters (Figure S4E). Because the mean is a poor statistic when cluster sample sizes are imbalanced, we calculated the SD of the silhouette coefficient (Figure S4E) and the entropy of the categorical distributions (by taking the sample ratios as parameters of the distribution) to evaluate class imbalance (Figure S4F). The elbow plot and difference of inertia are also shown to provide extra verification of the optimal k values' validity (Figure S4G). The optimal clustering results are shown as silhouette and scatterplots (Figures S4H and S4I) and interpreted as properties of clusters (based on coordination with biological relevance for each axis) (Figure S4J).

Although the clusters in the factor scores seemed to qualitatively correspond to the metadata for m1_10x, we used the conventional method to quantitively compare clustering performance. Dimensionality reduction via truncated SVD was performed, and the dimensionality of the output was determined via parallel analysis (Figure S5A); after clustering via SNN (Figure S5B), DEGs were identified by Wilcoxon rank-sum test (Figure S5C). Some mismatches occurred in the DEG expression patterns within a cell type (as given in the metadata for m1_10x); hence, the consistency of the partition (rather than the biological interpretation of clusters) was compared between the proposed and conventional clustering methods (Figures S5D and

Figure 5. A GBDT model to identify features with which to classify cell types in m1_10x

(A) A scatterplot of the uniform manifold approximation and projection (UMAP) manifold on expression patterns of the 90 marker genes in m1_10x. The marker colors denote cell types given in the metadata.

(B) Sample ratio transition during resampling and data splitting.

(C and D) SD among the group-wise mean values of gene expression.

(E) Subclass information given in the metadata of m1_10x.

(F) Top 10 genes (in terms of the median of FI) during 5-fold hyperparameter tuning.

(G and H) ROC curve and PR curve of the GBDT model with the top 1,000 genes (in terms of SD among the group-wise means). AUC, AP, and the macro or micro averages thereof were also calculated.

(I and J) The evaluation metrics for the GBDT model with *GAD1* and *GRIP1*.

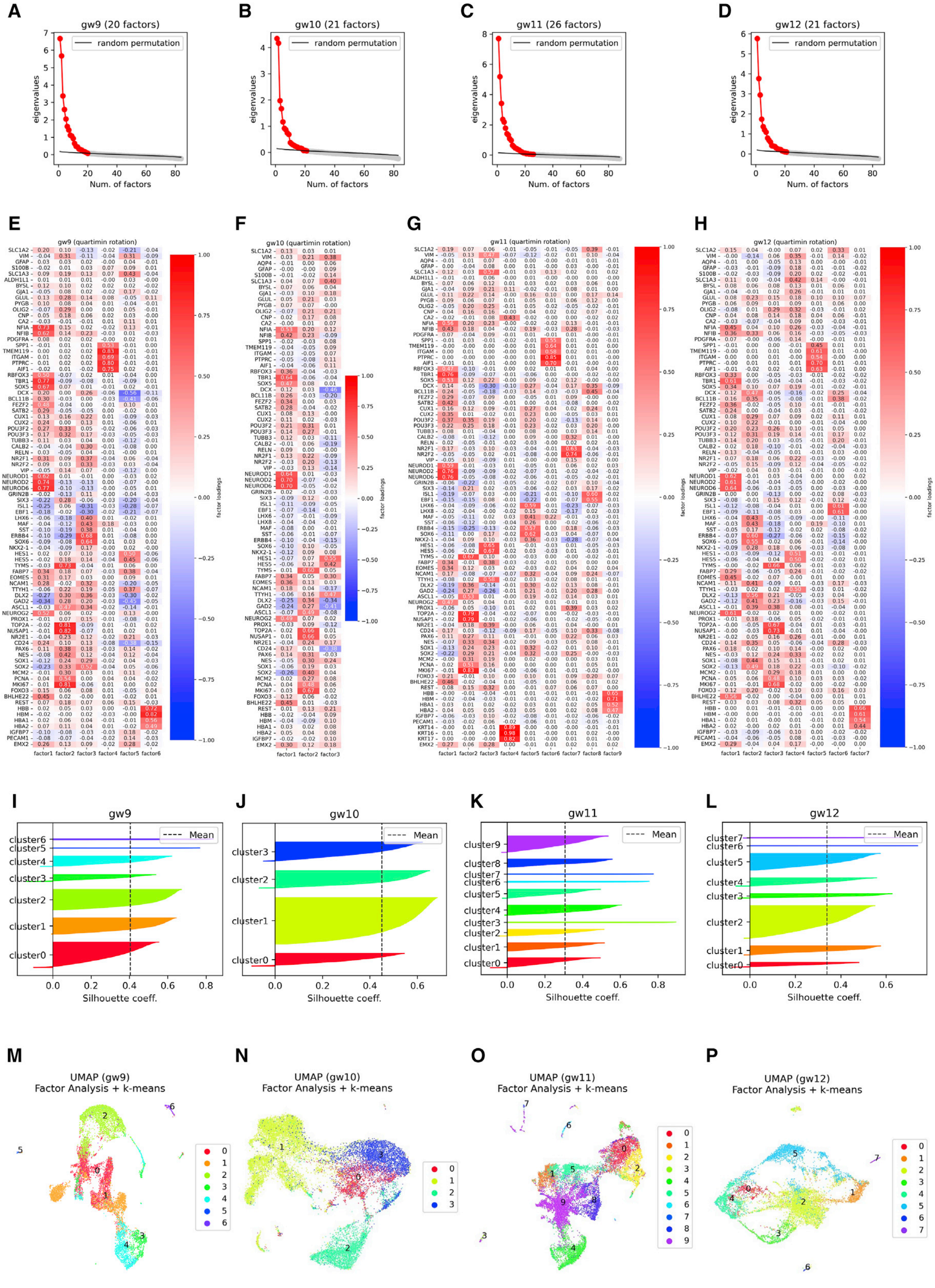

(legend on next page)

S5E). The factor analysis (FA) results showed a generally superior adjusted Rand index (ARI) and adjusted mutual information (AMI) scores compared with the conventional method (denoted as DEG); hence, we conclude that our method of clustering is more robust to changes in the sample ratio. Even though the higher AMI values for DEGs and subclasses suggest that the clustering of the conventional method was more similar to the subclass partitioning, we could not determine whether the clusters were similar to the subclasses because of the difference in DEG patterns. DEG patterns differ between clusters in m1_10x and gw9–gw12; hence, other approaches (besides computing DEGs) are required to compare the cellular identities of cell classes in different datasets.

FA and clustering for GSE165388 data

FA of the marker genes produced comprehensible and robust results; hence, we adopted the same procedure for GSE165388 before creating GRNs.

Variables were selected by referring to the KMO index and MSA, and the initial numbers of factors were determined via parallel analysis (Figures 6A–6D); then, final versions of the factor loading matrices for quartimin rotation (Figures 6E–6H) were obtained by recursively eliminating unnecessary factors. The communalities and uniquenesses of the variables were calculated for model evaluation (Figures S6A–S6D). After confirming that the marker genes for the same cellular identities showed similar factor loading values for each factor, k-means clustering was performed (Figures 6I–6L), and k values were selected after multiple verifications using the mean/SD of the silhouette coefficients, the entropy of the categorical distributions, and elbow plots (Figures S6E–S6P). The results are shown as scatterplots (Figures 6M−6P), and we qualitatively checked the biological properties of the clusters by verifying their coordinates (Figures S6Q–S6T); in general, no contradictions of the DEG results were observed.

GRNs and annotation methods based on their structural similarities

We implemented GRNs by applying the PC algorithm and compared their structural similarities using the evaluation function d^* proposed above. We implemented labeling (annotation-centering cell classes in the referential data) and estimation (annotation-centering cell classes in the query data) using the given GRNs.

For vertexes in GRNs, we selected genes with communalities exceeding 0.5 (*AQP4*, *GFAP*, *GJA1*, *PDGFRA*, *RBFOX3*, *SATB2*, *CUX2*, *VIP*, *GRIN2B*, *SOX6*, *NCAM1*, and *GAD2*) in the FA for m1_10x as well as the important features of the GBDT model (*GAD1* and *GRIP1*), anticipating that (1) genes with high communalities would express essences of the FA model that inherited biological functionalities and (2) the important features in the ML model would play crucial roles in discerning CNS cells. When labeling, we limited the vertexes used for comparison with cell type-specific marker genes as well as the important features of the ML model (Figure 7A). In contrast, we used all vertexes in estimation because the identities of the centered cell classes were unknown and we needed to match the vertexes to compare GRN structures (Figure S7A). We used appropriate induced subgraphs for labeling, although the GRNs are depicted with all genes in Figures 7B–7E.

For visualization of the GRN structural similarities, we present circular plots with focused cell classes in the centers of the circles; the d^* values are shown as the radii of the circles (Figures 7F–7H and S7B–S7E). We refer to these as "planet plots" because they resemble the solar system. We defined the labeling or estimation as a process of choosing cell classes that minimize d^*. Cell classes on the innermost circle were considered most similar to the cell class in the center of the plots.

The goal of annotation was to find GABAergic and glutamatergic neurons in GSE165388. When we aimed to select cell classes in the query data that were most similar to GABAergic or glutamatergic neurons in the referential data, labeling was suitable. When the aim was to choose a suitable name from GABAergic and glutamatergic neurons for each cluster in the query data, estimation was preferable.

The validity of the labeling results varied according to which cell class was centered. Assuming the characters of cell classes in the query data from the DEG and factor scores, NSCs and NPCs were frequently chosen as the cell classes most similar to the GABAergic neurons in the referential data (Figure 7A). The results for glutamatergic neurons seemed more promising because NSCs, NPCs, and excitatory neurons were frequently suggested (Figure 7B). Regarding non-neuronal cells, the results were meaningless because a variety of cell classes were suggested as similar

Figure 6. and k-means in GSE165388
(A–D) The parallel analyses used to determine the number of factors in gw9–gw12.
(E–H) Heatmaps of factor loadings after elimination of factors with maximum loadings smaller than 0.5. Quartimin rotation was performed for all models.
(I–L) Silhouette plots for optimal k values.
(M−P) Results of k-means clustering in UMAP.

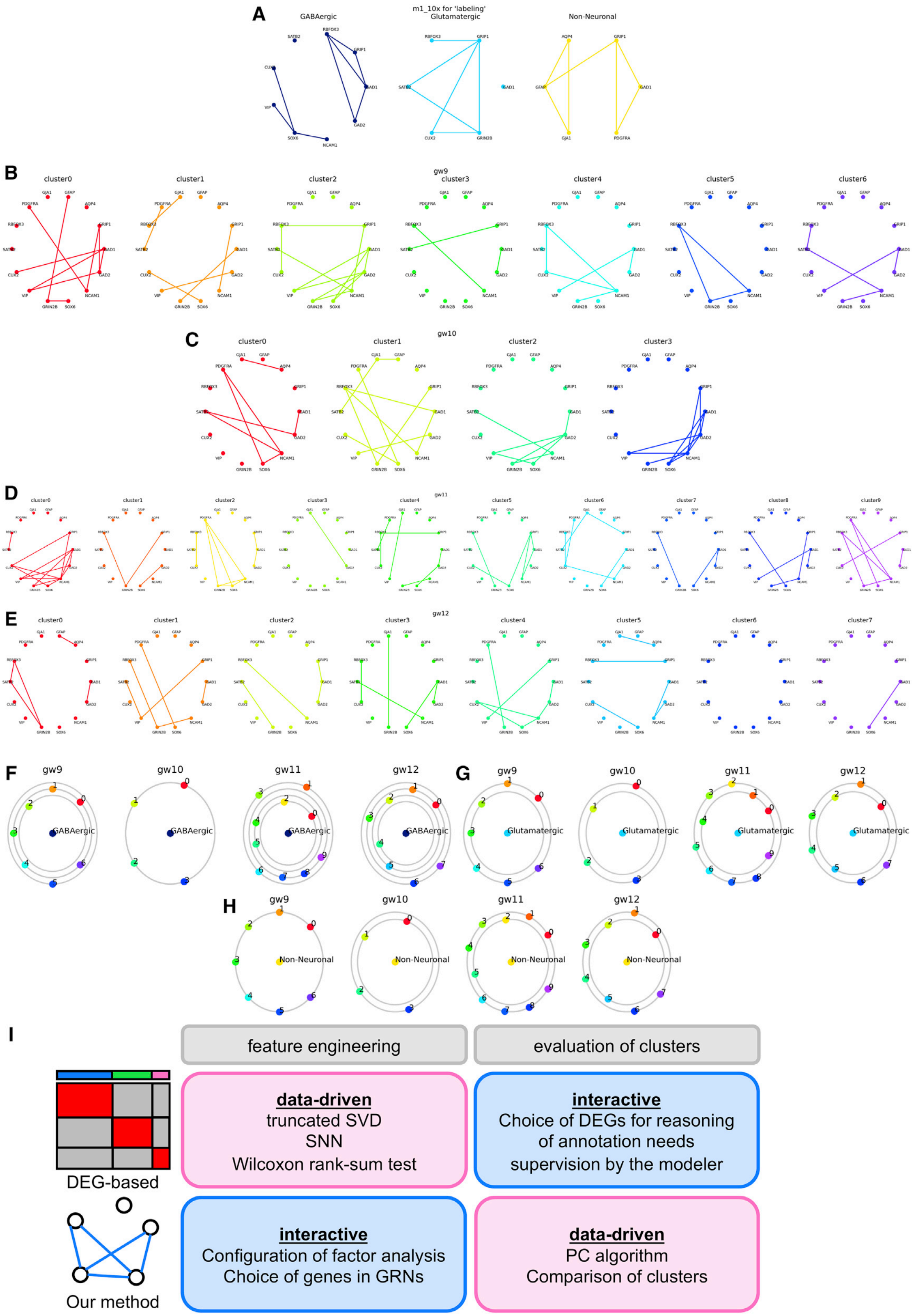

(legend on next page)

thereto. The imbalanced comparison itself might be problematic in that either class exhibits components of much greater diversity than the other one, such as GABAergic neurons versus non-neuronal CNS cells because, in such a case, similar and incompatible features can exist simultaneously in the larger class (Figure S7F).

The estimation performance was generally better than the labeling one: for inhibitory neurons (CGE, LGE, and MGE), GABAergic neurons or GABAergic plus non-neuronal linages were suggested; for excitatory neurons, glutamatergic neurons were selected (Figures S7A–S7D). In some cases that were difficult to determine (e.g., conjugated clusters of excitatory and inhibitory neurons or a cell class of NSCs), GABAergic and glutamatergic neurons were selected. The problem for non-neuronal cells also arose in estimation because these cells were in the innermost circles in many cases; however, the cell class was useful for estimating the cell classes of microglia and non-neuronal linages (i.e., microglia or RBCs).

Consequently, GRN-based annotation allowed us to compare the identities of cell classes by visualizing canonical concepts and properties shared between different datasets.

DISCUSSION

In this research, we developed a cellular identity representation that can be compared beyond the batch effects of different datasets, and we added mathematical descriptions to make the new concept more generally applicable. This can introduce quantitative elements into the empirical perspective of Linnean taxonomies while also adhering to biologists' belief of a certain universality in cell types. Numerical features of gene expressions can be diverse in high-dimensional matrices even when the biological semantics coincide; hence, we focused instead on the interactions of genes because we believe the latent mechanisms of a phenomenon to be reproductive in natural science. We also excluded variables that domain-specific knowledge cannot explain. Therefore, we needed to engage with the model to find the background structures of the data. When the model was established, we tried to remain unbiased during the deterministic procedure of annotation; hence, we selected a data-driven method. DEG-based annotation identifies features in data-driven ways and requires expert supervision during the deterministic process; our proposal does the opposite (Figure 7I). We emphasize that we can combine DEGs with GRNs and vice versa. DEG-based annotation is superior to our method of identifying unexpected features in data, and GRN comparisons are good at identifying shared information regarding cell classes. Because their domains of responsibility differ, they are not always mutual alternatives.

As noted above, GRN structure comparisons between different scales of categories can fail. We need to specify an evenly scaled control for adequate comparison, even when differences exist between them. This represents a fairly general restriction when comparing multiple things, not only in biological research; for instance, we cannot critically compare the city of Paris and the entire region of Asia without designating a representative city.

Several limitations were noted, as follows:

1. The choice of the algorithm. We used the PC algorithm to reduce computational costs; this suffers from a drawback, and several alternatives are available. The PC algorithm can be unstable when removing edges from the graph; to improve result reliability, the procedure of generating the graph should be supplemented with an algorithm to repair disconnected edges (Spirtes et al., 2000). A promising alternative is max-min hill-climbing (MMHC), which combines constraint-based local learning and scoring (Tsamardinos et al., 2006). MMHC is attractive because it can, in general, outperform other algorithms in practical tasks and is a down-to-earth option already available in specific tools such as Pgmpy.
2. Uncertainty in the hypothesis. We defined a model to handle cellular identities by starting the discussion with hypotheses yet to be proven. Outside of our model, the definition of a cell type has been controversial ever since single-cell technologies began to provide detailed information about cellular status. A paradigm shift has emerged in the classic notions of cell types, which are poorly defined but functional taxonomies (Clevers et al., 2017). To contribute to a solution to the debate, we strictly re-defined cellular identities as cell classes on hypothetical GRN models. Our model assumes that the cellular identity can be discussed using the dependencies and independencies of genes, whereas the innate GEN varies

Figure 7. GRN-based annotation in GSE165388
(A) GRNs of cell types in m1_10x to be used in labeling (as the referential data).
(B–E) GRNs of all clusters in gw9–gw12. All vertexes and edges used in labeling or estimation are indicated.
(F–H) "Planet plots" showing the labeling results. The referential cell classes are shown in the center, and the circles' radii denote values of d^*. The cell classes on the innermost circles are considered to be most similar to those in the center.
(I) Comparison of DEG- and GRN-based annotation methods.

under observation. Hence, further discussions and experimental proof are required.

3. Time resolution. Analysis of non-time-series RNA-seq data suffers from limitations, and the new methods we proposed in this study also exhibit the same drawbacks. It is nearly impossible to perfectly identify the cellular dynamics of every single cell using a snapshot of cells'' multifaceted aspects. At best, we could assume no fluctuation in experimental conditions and assume that the model can track temporal changes by tracing samples using continuous curves. Under definitions of cell class and eigen-cascades, heterogeneous cell populations can be divided. Hence, we require a nontrivial operation to collect multiple cell classes into one unit when treating them as identical a group of cells that dynamically drift around the "attractor" of the cellular lineage (e.g., each phase of the cell cycle).

Although several issues remain, GRNs and the concept of cell classes are applicable in certain studies pertaining to the canonical state (e.g., patient-derived samples) and simplified models (e.g., cultured cells, model animals, etc.) because cell class annotation compares the structures of GRNs but not the raw values of the expression. This will help scientists to explicitly describe what aspects of the cells in their samples are universal.

EXPERIMENTAL PROCEDURES

Resource availability

Corresponding author

Further information and requests for resources and reagents should be directed to and will be fulfilled by the corresponding author, H.O. (hidokano@keio.jp).

Materials availability

Not applicable.

Data and code availability

All of the code we used has already been deposited in a GitHub repository (https://github.com/yo-aka-gene/algebra_ver822), and the sources are listed in the Experimental procedures section and in the figure legends.

Data preprocessing of adult human brain samples

The scRNA-seq data for an adult human brain were downloaded from the website of the Allen Institute for Brain Science (https://portal.brain-map.org/atlases-anddata/rnaseq/human-m1-10x), and we normalized the expression matrix into read per million (RPM) and converted it further into log2(RPM+1). The code was implemented in Python packages (Numpy and Pandas).

Data preprocessing of fetal human brain samples

The scRNA-seq datasets for fetal human brains (GSE165388) were downloaded from https://www.ncbi.nlm.nih.gov/geo/query/acc.cgi?acc=GSE165388 and processed. Quality control using mitochondrial genes and ln(RPM+1) conversion were performed. The code was implemented in an R package (Seurat).

SUPPLEMENTAL INFORMATION

Supplemental information can be found online at https://doi.org/10.1016/j.stemcr.2022.10.015.

AUTHOR CONTRIBUTIONS

Y.O. obtained funding, designed the model, performed the experiments, analyzed the results, and wrote the paper. Y.K. obtained funding and edited the paper as an instructor. H.O. obtained funding, edited the paper, and supervised the project. All authors approved the final manuscript.

ACKNOWLEDGMENTS

We thank Prof. Kazuhiro Sakurada and Dr. Tetsuo Ishikawa (Department of Extended Intelligence for Medicine, The Ishii-Ishibashi Laboratory, Keio University School of Medicine) for critiquing the manuscript. This work was supported by the Keio University Medical Science Fund (to Y.O.), the General Insurance Association of Japan (to Y.K.), the Takeda Science Foundation (to Y.K.), JSPS KAKENHI grant 22K16696 (to Y.K.), the Next-Generation Research Project Promotion Program of Keio University (to H.O.), and the Yagami Data Security Lab Project of Keio University.

CONFLICT OF INTERESTS

H.O. is a compensated scientific consultant for San Bio Co., Ltd.; RMiC; and K Pharma, Inc.

Received: April 9, 2022
Revised: October 19, 2022
Accepted: October 20, 2022
Published: November 17, 2022

REFERENCES

Aalto, A., Viitasaari, L., Ilmonen, P., Mombaerts, L., and Gonçalves, J. (2020). Gene regulatory network inference from sparsely sampled noisy data. Nat. Commun. *11*, 3493.

Allen, P.J. (1969). A fundamental theorem of homomorphisms for semirings. Proc. Am. Math. Soc. *21*, 412–416.

Ankan, A., and Panda, A. (2015). Pgmpy: probabilistic graphical models using Python. In Proc. 14th Python Sci. Conf. https://doi.org/10.25080/MAJORA-7B98E3ED-001.

Bookstein, A., Kulyukin, V.A., and Raita, T. (2002). Generalized hamming distance. Inf. Retr. Boston. *5*, 353–375.

Cheng, J., Bell, D.a., and Liu, W. (1997). An algorithm for Bayesian belief network construction from data. Proc. Mach. Learn. Res., 83–90.

Clevers, H., Rafelski, S., Elowitz, M., Klein, A., Shendure, J., Trapnell, C., Lein, E., Lundberg, E., Uhlen, M., Martinez-Arias, A., et al. (2017). What is your conceptual definition of "cell type" in the context of a mature organism? Cell Syst. *4*, 255–259.

Edelhoff, S., Grubin, C.E., Karlsen, A.E., Alder, D.A., Foster, D., Disteche, C.M., and Lernmark, Å. (1993). Mapping of glutamic acid decarboxylase (GAD) genes. Genomics *17*, 93–97.

Grigg, N., and Manwaring, N. (2007). An elementary proof of the fundamental theorem of tropical Algebra. Preprint at arXiv. https://doi.org/10.48550/arXiv.0707.2591.

Herrlich, H., and Keremedis, K. (2015). On the metric reflection of a pseudometric space in ZF. Comment. Math. Univ. Carol. *56*, 77–88.

Jain, V., Yang, W.H., Wu, J., Roback, J.D., Gregory, S.G., and Chi, J.T. (2022). Single cell RNA-seq analysis of human red cells. Front. Physiol. *13*, 828700.

Katz, E. (2017). WHAT IS...tropical geometry? Not. Am. Math. Soc. *64*, 380–382.

Kiselev, V.Y., Andrews, T.S., and Hemberg, M. (2019). Challenges in unsupervised clustering of single-cell RNA-seq data. Nat. Rev. Genet. *20*, 273–282.

Kũnzi, H.P. (1992). Complete quasi-pseudo-metric spaces. Acta Math. Hung. *59*, 121–146.

Ledesma, R.D., and Valero-Mora, P. (2007). Determining the number of factors to retain in EFA: an easy-to-use computer program for carrying out Parallel Analysis. Pract. Assessment, Res. Eval. *12*.

Ledesma, R.D., Valero-Mora, P., and Macbeth, G. (2015). The scree test and the number of factors: a dynamic graphics approach. Span. J. Psychol. *18*, E11.

Lier, J., Streit, W.J., and Bechmann, I. (2021). Beyond activation: characterizing microglial functional phenotypes. Cells *10*, 2236.

Lu, C., Shi, X., Allen, A., Baez-Nieto, D., Nikish, A., Sanjana, N.E., and Pan, J.Q. (2019). Overexpression of NEUROG2 and NEUROG1 in human embryonic stem cells produces a network of excitatory and inhibitory neurons. FASEB J. *33*, 5287–5299.

Manetti, M. (2015). Topology (Springer).

Mitchell, B.S.S., and Sinutoke, P. (1982). The theory of semifields. Kyungpook Math. J. *22*, 325–348.

Murthy, S., Niquille, M., Hurni, N., Limoni, G., Frazer, S., Chameau, P., Van Hooft, J.A., Vitalis, T., and Dayer, A. (2014). Serotonin receptor 3A controls interneuron migration into the neocortex. Nat. Commun. *5*, 5524.

Pasquini, G., Rojo Arias, J.E., Schäfer, P., and Busskamp, V. (2021). Automated methods for cell type annotation on scRNA-seq data. Comput. Struct. Biotechnol. J. *19*, 961–969.

Pearl, J. (2009). Causal inference in statistics: an overview. Stat. Surv. *3*, 96–146.

Pin, J.-E. (2010). Tropical semirings. Idempotency, 50–69.

Simon, I. (1978). Limited subsets of a free monoid. In Proceedings - Annual Symposium on Foundations of Computer Science, 1978 (IEEE), pp. 143–150.

Spirtes, P., Glymour, C., and Scheines, R. (2000). Causation, Prediction, and Search (The MIT press).

Street, K., Risso, D., Fletcher, R.B., Das, D., Ngai, J., Yosef, N., Purdom, E., and Dudoit, S. (2018). Slingshot: cell lineage and pseudotime inference for single-cell transcriptomics. BMC Genom. *19*, 477.

Tan, H.L., Chiu, S.L., Zhu, Q., and Huganir, R.L. (2020). GRIP1 regulates synaptic plasticity and learning and memory. Proc. Natl. Acad. Sci. USA *117*, 25085–25091.

Tanay, A., and Sebé-Pedrós, A. (2021). Evolutionary cell type mapping with single-cell genomics. Trends Genet. *37*, 919–932.

Tsamardinos, I., Brown, L.E., and Aliferis, C.F. (2006). The max-min hill-climbing Bayesian network structure learning algorithm. Mach. Learn. *65*, 31–78.

de Vargas Roditi, L., and Claassen, M. (2015). Computational and experimental single cell biology techniques for the definition of cell type heterogeneity, interplay and intracellular dynamics. Curr. Opin. Biotechnol. *34*, 9–15.

Wright-Jin, E.C., and Gutmann, D.H. (2019). Microglia as dynamic cellular mediators of brain function. Trends Mol. Med. *25*, 967–979.

Yu, Y., Zeng, Z., Xie, D., Chen, R., Sha, Y., Huang, S., Cai, W., Chen, W., Li, W., Ke, R., et al. (2021). Interneuron origin and molecular diversity in the human fetal brain. Nat. Neurosci. *24*, 1745–1756.

Zhang, J., and Jiao, J. (2015). Molecular biomarkers for embryonic and adult neural stem cell and neurogenesis. BioMed Res. Int. *2015*, 727542.

Zumbrägel, J., and Zelmanov, E. (2008). Classification of finite congruence-simple semirings with zero. J. Algebra Appl. *07*, 363–377.

Stem Cell Reports, Volume *18*

Supplemental Information

A set-theoretic definition of cell types with an algebraic structure on gene regulatory networks and application in annotation of RNA-seq data

Yuji Okano, Yoshitaka Kase, and Hideyuki Okano

Supplemental Experimental Procedures

Supplemental Methods

Tropical geometry and tropical semirings

Tropical geometry is considered over the classical tropical semifield $(\mathbb{R} \cup \{\infty\}, \oplus, \odot)$ defined as follows (Katz, 2017):

$$\forall a, b \in \mathbb{R} \cup \{\infty\}$$
$$a \oplus b = \min(a, b)$$
$$a \odot b = a + b$$

Note that a semifield $(K, +, \cdot)$ is a semiring such that $(K, \cdot)$ is an abelian group with the zero of the semifield K (i.e., an absorbing element of multiplication $a \in K$ such that $(K - \{a\}, \cdot)$ forms an abelian group (Mitchell and Sinutoke, 1982)); therefore, by definition, tropical semifields always fulfill the definition of a semiring. Other tropical analogs can be defined as well (Katz, 2017).

Commutative semiring

When $(\{0, 1, \infty\}, \oplus, \odot)$ is a commutative semiring (consider addition and multiplication to be defined as well as the tropical semiring), it fulfills all the following requirements (Pin, 2010; Zumbrägel and Zelmanov, 2008).

1. $\{0, 1, \infty\}$ is a commutative monoid for the addition (in this case, $\oplus$), with the neutral

element called $zero$ (in this case, ∞).

2. $\{0, 1, \infty\}$ is a monoid for the multiplication (in this case, $\odot$), with the neutral element called one (in this case, 0).

3. $\forall a, b, c, \in \{0, 1, \infty\}\ s.t.\ a(b \oplus c) = ab \oplus ac\ \wedge (a \oplus b)c = ac \oplus bc$ (i.e., multiplication is distributive over addition).

4. $\forall a \in \{0, 1, \infty\}, \exists! \infty \in \{0, 1, \infty\}, a \oplus \varepsilon = \varepsilon \oplus a = a\ s.t.\ a \odot \varepsilon = \varepsilon \odot a = \varepsilon$ (i.e., the neutral element for the addition is an absorbing element for the multiplication).

5. Multiplication is commutative.

Quotient semiring and natural homomorphism

Suppose $\forall (R, +, \cdot), (S, +, \cdot): Semirings, \forall f: R \rightarrow S\ s.t.\ \forall r_1, r_2 \in R, f(r_1 + r_2) = f(r_1) + f(r_2) \wedge f(r_1 r_2) = f(r_1) \cdot f(r_2)$, where f is the map that preserves the semiring operations and the zero element (hence defined as a semiring homomorphism) (Zumbrägel and Zelmanov, 2008). Moreover, we considered an equivalence relation $\sim$ on a semiring $(R, +, \cdot)$ as congruence where the notation $r_1 \sim r_2$ implies $r_1 + s \sim r_2 + s \wedge sr_1 \sim sr_2 \wedge r_1 s \sim r_2 s$ for all $r_1, r_2, s \in R$ (Zumbrägel and Zelmanov, 2008). When these definitions for a homomorphism and congruence are given, we can define the same operations ($+$ and $\cdot$) as R on its set of equivalence classes $R/{\sim} = \{[r_1] | r_1 \in R\}$

by $[r_1]+[r_2]\coloneqq[r_1+r_2]$ and $[r_1]\cdot[r_2]\coloneqq[r_1r_2]$ for all $r_1,r_2\in R$ (an equivalent class is denoted as $[r_1]\coloneqq\{r_2\in R|r_1\sim r_2\}$ and $(R/{\sim},+,\cdot)$ is called the quotient semiring). In addition, the map $\pi: R\to R/{\sim}$ used to generate the quotient semiring of R always satisfies congruence $\sim$ on R, because we define $r_1\sim r_2$ if and only if $\pi(r_1)=\pi(r_2)$ for all $r_1,r_2\in R$; thus, π is called the natural homomorphism $R\to R/{\sim}$ (Zumbrägel and Zelmanov, 2008).

Ideal on a semiring

The definition of an ideal I_R on a semiring $(R,+,\cdot)$ is given as follows (Allen, 1969):

$$\forall I_R\subset R,\forall i,j\in I_R,\forall r\in R\ \ s.t.\ \ i+j\in I_R\ \wedge\ r\cdot i\in I_R\ \wedge\ i\cdot r\in I_R.$$

When I_R is an ideal of R, $[r]=r+I_R\coloneqq\{r+i|i\in I_R\}$ for all $r\in R$. Hence, the design of the ideals determines the configuration of the equivalent relation ($\forall r\in R,\forall i\in I_R\ s.t.\ r\sim r+i$) and corresponding equivalent classes. Note that $(R/I_R,+,\cdot)$ is a quotient semiring, where $R/I_R=\{[r]|r\in R\}$.

Hamming Distance

The Hamming distance is frequently used to quantify the difference between two bitstrings of the same dimension (i.e., length) (Bookstein et al., 2002). It is defined as the

number of mismatches in the corresponding bits of the two strings. The three strings 100010, 010010, and 001010 all differ from each other by a Hamming distance of 2 (Bookstein et al., 2002).

Topological space and open ball

A topology on a set X is defined as a family $\mathcal{O}$ of subsets of X (called open sets) where $\mathcal{O}$ satisfies the following:

1. $\emptyset \subset \mathcal{O} \wedge X \subset \mathcal{O}$.

2. $\forall \Lambda\text{: Set}, \forall \lambda \in \Lambda,\ o_\lambda \in \mathcal{O}\ s.t. \bigcup_{\lambda \in \Lambda} o_\lambda \in \mathcal{O}$.

3. $\forall O_1, O_2 \in \mathcal{O}\ s.t. O_1 \cap O_2 \in \mathcal{O}$.

When a set is equipped with a topology, it is called a topological space. In terms of X and $\mathcal{O}$, $(X, \mathcal{O})$ is a topological space (Manetti, 2015).

The definition of the open ball requires a distance function; hence, we verify this here. A distance on a set X is a function $d: X \times X \to \mathbb{R}$ that satisfies the following criteria:

1. $\forall x, y \in X, d(x, y) \geq 0 \wedge d(x, y) = 0 \Leftrightarrow x = y$.

2. $\forall x, y \in X, d(x, y) = d(y, x)$.

3. $\forall x, y, z \in X, d(x, y) + d(y, z) \geq d(x, z)$.

Note that a metric space is a pair (X, d) consisting of a set X and a distance d on X. The topology of the metric space (called the metric topology) is determined by the distance and described using open balls. An open ball $B(x, r)$ centered at x with radius r is a subset of the metric space defined with parameters $x, r \in \mathbb{R}$ as

$$B(x, r) \coloneqq \{y \in X | d(x, y) < r\}.$$

Note that $B_r(x)$ is an equivalent notation, which is being curried about r. Given the definition of an open ball, the metric topology $\mathcal{T}$ is defined as follows:

$$\mathcal{T} \coloneqq \{T \subset X | \forall x \in T, \exists r \in \mathbb{R}_{>0}\ s.t. B(x, r) \subset T\},$$

where $\mathbb{R}_{>0}$ is a positive subset of real numbers. The definition of metric topology satisfies the definition of topology (Manetti, 2015).

Quasi-pseudo-metric, pseudo-metric, and topology of pseudo-metric space

A quasi-pseudo-metric p is a mapping $p: X \times X \to \mathbb{R}$, where X is a set and p satisfies the following (Kũnzi, 1992):

1. $\forall x, y \in X, p(x, y) \geq 0.$

2. $\forall x \in X, p(x, x) = 0.$

3. $\forall x, y, z \in X, p(x, y) + p(y, x) \geq p(x, z).$

A pseudo-metric ρ is a mapping $\rho: X \times X \to \mathbb{R}$, where X is a set and p satisfies

the following (Herrlich and Keremedis, 2015):

1. $\forall x, y \in X, \rho(x, y) \geq 0 \wedge \rho(x, x) = 0$.

2. $\forall x, y \in X, \rho(x, y) = \rho(y, x)$.

3. $\forall x, y, z \in X, \rho(x, y) + \rho(y, z) \geq \rho(x, z)$.

Given the definition of pseudo-metric, (X, ρ) is a pseudo-metric space. Pseudo-metric topology is also generated by an open ball $D(x, \epsilon)$, defined as

$$D(x, \epsilon) = \{y \in X | \rho(x, y) < \epsilon\} .$$

When we introduce an equivalence relation $\sim$ on X (given by $x \sim y$ if and only if $\rho(x, y) = 0$), the quotient space (X^*, ρ^*) of (X, ρ) is a metric space because the metric reflection is introduced by $\sim$, where X^* is the set of all equivalent classes in X and $\rho^*: X^* \times X^* \to \mathbb{R}$ is given by the following; meanwhile, $[x]$ is the equivalent class of $x \in X$ (Herrlich and Keremedis, 2015), such that

$$\rho^*([x], [y]) = \rho(x, y).$$

Method details

Under-sampling

Random under-sampling was performed to resolve the class imbalance in m1_10x. Samples were stratified according to the metadata; then, resampling was performed to make the sample sizes of all cell types even numbers. The resampled data were used for GBDT, factor analysis, DEG-based manual annotation, and GRN-based annotation. The code was implemented in Python packages (Imbalanced-learn, Numpy, and Pandas).

Data splitting

To prevent data leakage, groups were generated for the "GroupShuffleSplit" method in Scikit-learn. The expression matrix for the 90 marker genes in the resampled m1_10x were converted into a matrix with lower dimensionality, via principal component analysis (PCA); meanwhile, the number of components was automatically determined (Minka, 2001), then, samples were classified into 500 clusters using k-means algorithm. Next, the resampled data were randomly split into training/test data (4:1) using the GroupShuffleSplit method. The random seed was fixed throughout the process. The

code was implemented using a Python package (Scikit-learn).

Factor analysis

Factor analysis using quartimin rotation was performed. The initial number of factors was determined according to parallel analysis with null models of randomly permutated data matrices. After quartimin rotation, if the model included factors with maximum absolute factor loading values smaller than 0.5, parallel analysis was performed on the factor score matrices and a reduced number of factors was obtained. These processes were iteratively performed whilst factors with small loadings were retained in the model. The code was implemented using Python packages (Factor_analyzer, Matplotlib, Numpy, Pandas, and Seaborn).

K-means clustering

K-means clustering was performed. The optimal number of clusters was determined by the mean silhouette scores. The random seed was fixed throughout the process. The code was implemented in Python packages (Matplotlib, Pandas, Scikit-learn, and Seaborn).

SNN clustering

In the DEG-based annotation, clustering was performed using the “FindClusters” method with resolution of 0.8. Other arguments were fixed as default values. The code was performed using an R package (Seurat).

Identification of variable features and DEGs

For gw9–gw12, 2000 variable features were identified for each using “FindVariableFeatures” method with default arguments. DEGs were identified for each cluster using “FindAllMakers” method. The threshold for log FC was set to 0.25. The code was implemented using an R package (Seurat).

Dimensionality reduction (truncated SVD and UMAP)

The data dimensionality was determined via parallel analysis for the explained variance calculated using truncated SVD. The data were sent for further analysis once the decomposed matrices had been cropped to match the dimensionality of the parallel analysis. For gw9–gw12, the matrices of lower dimensionality were embedded into the UMAP manifold using the “RunUMAP” method. For m1_10x, after PCA dimensionality reduction, the UMAP method was used to embed the matrix. The codes were

implemented in R packages (Seurat, Dplyr, and Jsonlite) and Python packages (Matplotlib, Pandas, Scikit-learn, Scipy, Seaborn, and Umap-learn).

GBDT

GBDT with L1 and L2 regularizations was selected as an ML model for multiclass classification. To reduce the computational costs, the 1000 genes with the highest S.D. amongst the group-wise mean values were selected as features. Hyperparameter tuning was performed using five-fold cross validation with logarithmic loss chosen as the objective function. The FI was calculated in each fold. The prediction performance of the model with optimized hyperparameters was verified on the test data according to multiple indices (AUC of ROC curve, AP, macro average of AUC, and micro average of AP). To ensure that the important features adequately explained the classification, a new GBDT model containing the important features was constructed via the same process. The code was implemented using Python packages (LightGBM, Matplolib, Numpy, Optuna, Pandas, Scikit-learn, and Seaborn).

PC algorithm and GRN construction

The expression matrix of the genes exhibited high communalities in the factor analysis

for the resampled m1_10x and important features of the GBDT model were selected. DAGs were obtained using the PC algorithm with a test of uncorrelation in terms of Pearson's correlation coefficient (significance level: 0.01). To convert the DAGs into undirected GRNs (without ε), the directionalities of the edges were neglected. The edge information was converted into matrices by overwriting the corresponding elements in an $n \times n$ identity matrix with 1 if the edge existed in the GRN (where n is the number of vertexes). The code was implemented using Python packages (Numpy, Pandas, and Pgmpy).

Visualization of GRN-based annotation

The values of d^* were calculated from the matrices that storing GRN information. The cardinalities of the eigen-cascades were calculated as the sum of all elements in the matrix, and the intersections of the eigen-cascades were calculated as Hadamard products. Note that the diagonal components correspond to edges from ε to all vertexes. The code was implemented using Python packages (Matplolib, Numpy, and Pandas).

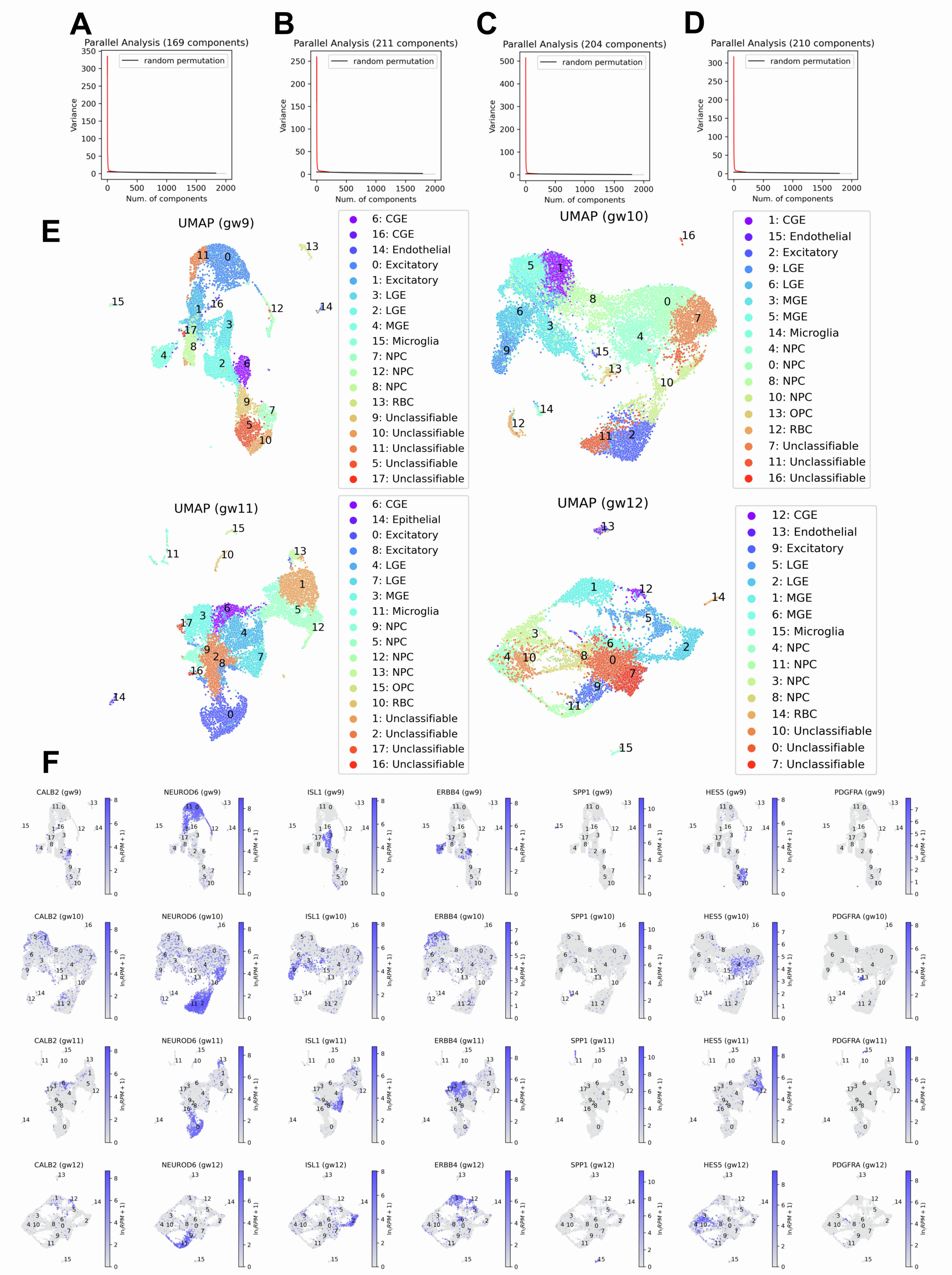

A
Parallel Analysis (169 components)
B
Parallel Analysis (211 components)
C
Parallel Analysis (204 components)
D
Parallel Analysis (210 components)
random permutation
Variance
Num. of components
E
UMAP (gw9)
6: CGE
16: CGE
14: Endothelial
0: Excitatory
1: Excitatory
3: LGE
2: LGE
4: MGE
15: Microglia
7: NPC
12: NPC
8: NPC
13: RBC
9: Unclassifiable
10: Unclassifiable
11: Unclassifiable
5: Unclassifiable
17: Unclassifiable
UMAP (gw10)
1: CGE
15: Endothelial
2: Excitatory
9: LGE
6: LGE
3: MGE
5: MGE
14: Microglia
4: NPC
0: NPC
8: NPC
10: NPC
13: OPC
12: RBC
7: Unclassifiable
11: Unclassifiable
16: Unclassifiable
UMAP (gw11)
6: CGE
14: Epithelial
0: Excitatory
8: Excitatory
4: LGE
7: LGE
3: MGE
11: Microglia
9: NPC
5: NPC
12: NPC
13: NPC
15: OPC
10: RBC
1: Unclassifiable
2: Unclassifiable
17: Unclassifiable
16: Unclassifiable
UMAP (gw12)
12: CGE
13: Endothelial
9: Excitatory
5: LGE
2: LGE
1: MGE
6: MGE
15: Microglia
4: NPC
11: NPC
3: NPC
8: NPC
14: RBC
10: Unclassifiable
0: Unclassifiable
7: Unclassifiable
F
CALB2 (gw9)
NEUROD6 (gw9)
ISL1 (gw9)
ERBB4 (gw9)
SPP1 (gw9)
HES5 (gw9)
PDGFRA (gw9)
CALB2 (gw10)
NEUROD6 (gw10)
ISL1 (gw10)
ERBB4 (gw10)
SPP1 (gw10)
HES5 (gw10)
PDGFRA (gw10)
CALB2 (gw11)
NEUROD6 (gw11)
ISL1 (gw11)
ERBB4 (gw11)
SPP1 (gw11)
HES5 (gw11)
PDGFRA (gw11)
CALB2 (gw12)
NEUROD6 (gw12)
ISL1 (gw12)
ERBB4 (gw12)
SPP1 (gw12)
HES5 (gw12)
PDGFRA (gw12)
ln(RPM + 1)

Figure S1

Figure S1. DEG-based annotation on GSE165388

(A–D) The parallel analyses used to determine the dimensionalities of variable features in gw9–gw12.

(E) Results of SNN clustering in UMAP. The clusters refer to the DEGs.

(F) The expression patterns of representative marker genes for their respective cell types.

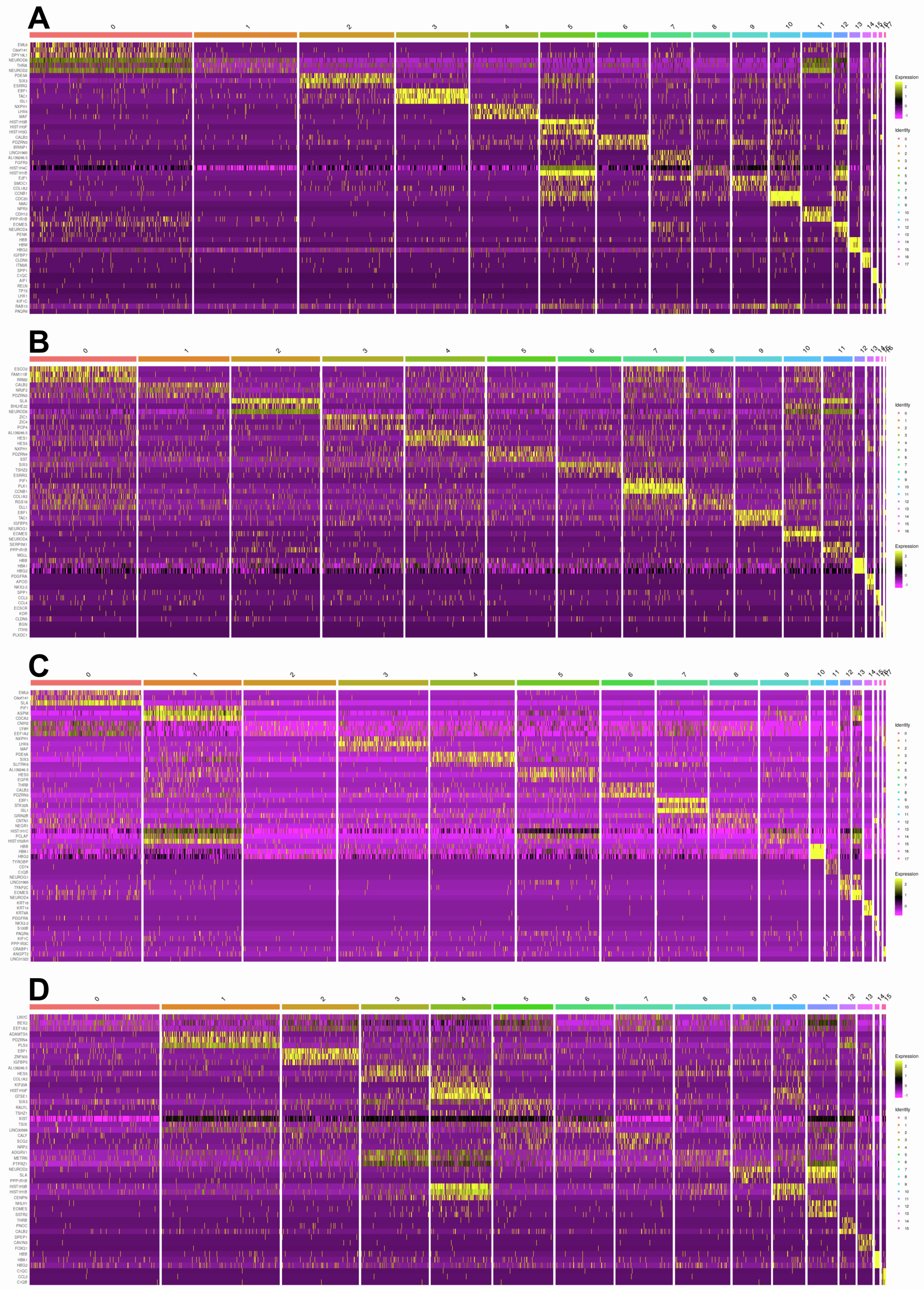

Figure S2

Figure S2. The expression patterns of DEGs, which are inconsistent even for the clusters under the same name.

(A–D) Heatmaps of the DEGs for their respective clusters. The Wilcoxon rank-sum test was performed to identify DEGs in (A) gw9, (B) gw10, (C) gw11, and (D) gw12. The top three genes with the largest log FC values are shown for each cluster.

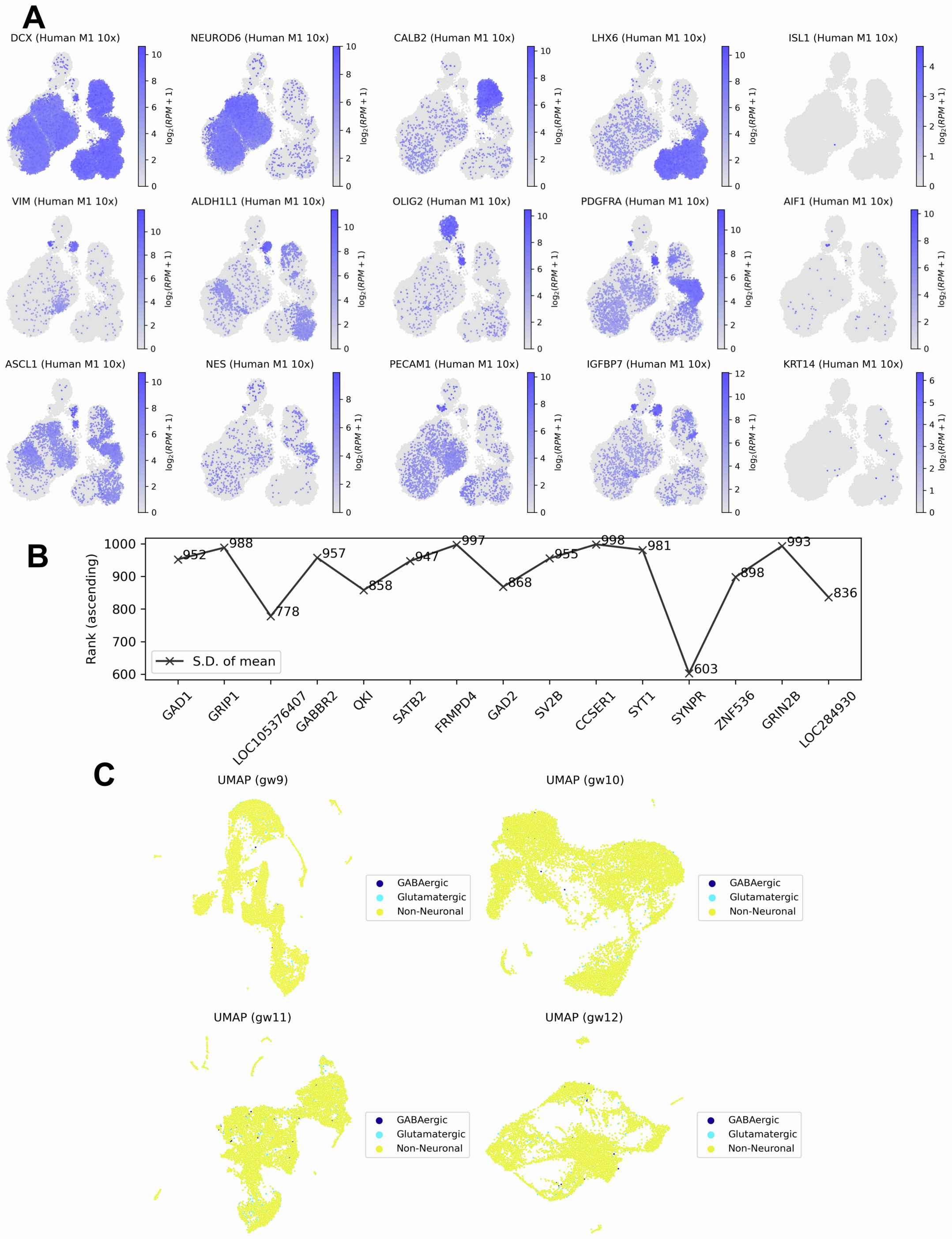
A
DCX (Human M1 10x)
NEUROD6 (Human M1 10x)
CALB2 (Human M1 10x)
LHX6 (Human M1 10x)
ISL1 (Human M1 10x)
VIM (Human M1 10x)
ALDH1L1 (Human M1 10x)
OLIG2 (Human M1 10x)
PDGFRA (Human M1 10x)
AIF1 (Human M1 10x)
ASCL1 (Human M1 10x)
NES (Human M1 10x)
PECAM1 (Human M1 10x)
IGFBP7 (Human M1 10x)
KRT14 (Human M1 10x)
log2(RPM + 1)
B
Rank (ascending)
S.D. of mean
GAD1
GRIP1
LOC105376407
GABBR2
QKI
SATB2
FRMPD4
GAD2
SV2B
CCSER1
SYT1
SYNPR
ZNF536
GRIN2B
LOC284930
952
988
778
957
858
947
997
868
955
998
981
603
898
993
836
C
UMAP (gw9)
UMAP (gw10)
UMAP (gw11)
UMAP (gw12)
GABAergic
Glutamatergic
Non-Neuronal

Figure S3

Figure S3. Supplemental information for the m1_10x and the GBDT model.

(A) Expression patterns of representative marker genes for their respective cell types.

(B) The relation between S.D. of group-wise mean values and FI for the top 15 genes (in terms of FI) in the GBDT model.

(C) Automatic annotation results obtained using the GBDT model. Our model was not designed for automatic annotation; hence, prediction performance was poor. The datasets from GSE165388 were converted into $\log_2$(RPM+1), to adjust the data scales in m1_10x before prediction.

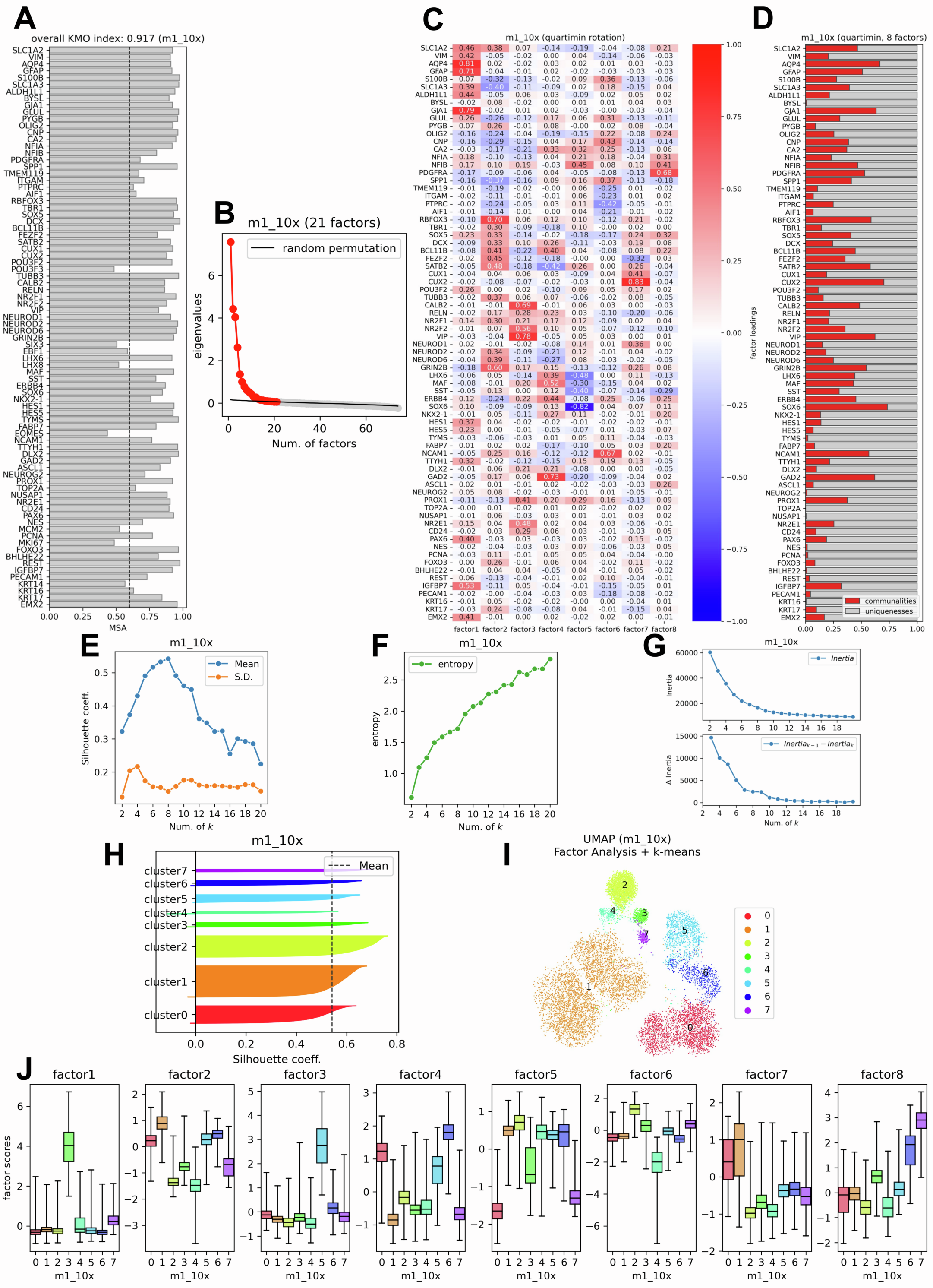
A
overall KMO index: 0.917 (m1_10x)
MSA
B
m1_10x (21 factors)
random permutation
eigenvalues
Num. of factors
C
m1_10x (quartimin rotation)
factor loadings
D
m1_10x (quartimin, 8 factors)
communalities
uniquenesses
E
m1_10x
Mean
S.D.
Silhouette coeff.
Num. of k
F
m1_10x
entropy
Num. of k
G
m1_10x
Inertia
Δ Inertia
Num. of k
H
m1_10x
Mean
cluster7
cluster6
cluster5
cluster4
cluster3
cluster2
cluster1
cluster0
Silhouette coeff.
I
UMAP (m1_10x)
Factor Analysis + k-means
J
factor1
factor2
factor3
factor4
factor5
factor6
factor7
factor8
factor scores
m1_10x

Figure S4

Figure S4. Factor analysis + k-means clustering in m1_10x.

(A) Overall KMO index and MSA values in each variable.

(B) Parallel analysis used to determine the number of factors.

(C) Heatmaps of factor loadings after eliminating factors with maximum loadings smaller than 0.5. Quartimin rotation was performed for all steps.

(D) Communalities and the uniquenesses for each variable in the model.

(E) Transition of the mean and S.D. of the silhouette coefficients.

(F) Transition of entropies for categorical distributions in which parameters were point-estimated using the sample size ratios of clusters.

(G) Elbow plot and the difference of inertia ($\Delta Inertia_k \coloneqq Inertia_{k-1} - Inertia_k$).

(H) Silhouette plot in the optimal k value.

(I) Result of k-means clustering in UMAP.

(J) Coordinates of clusters (corresponding to factors) in each axis.

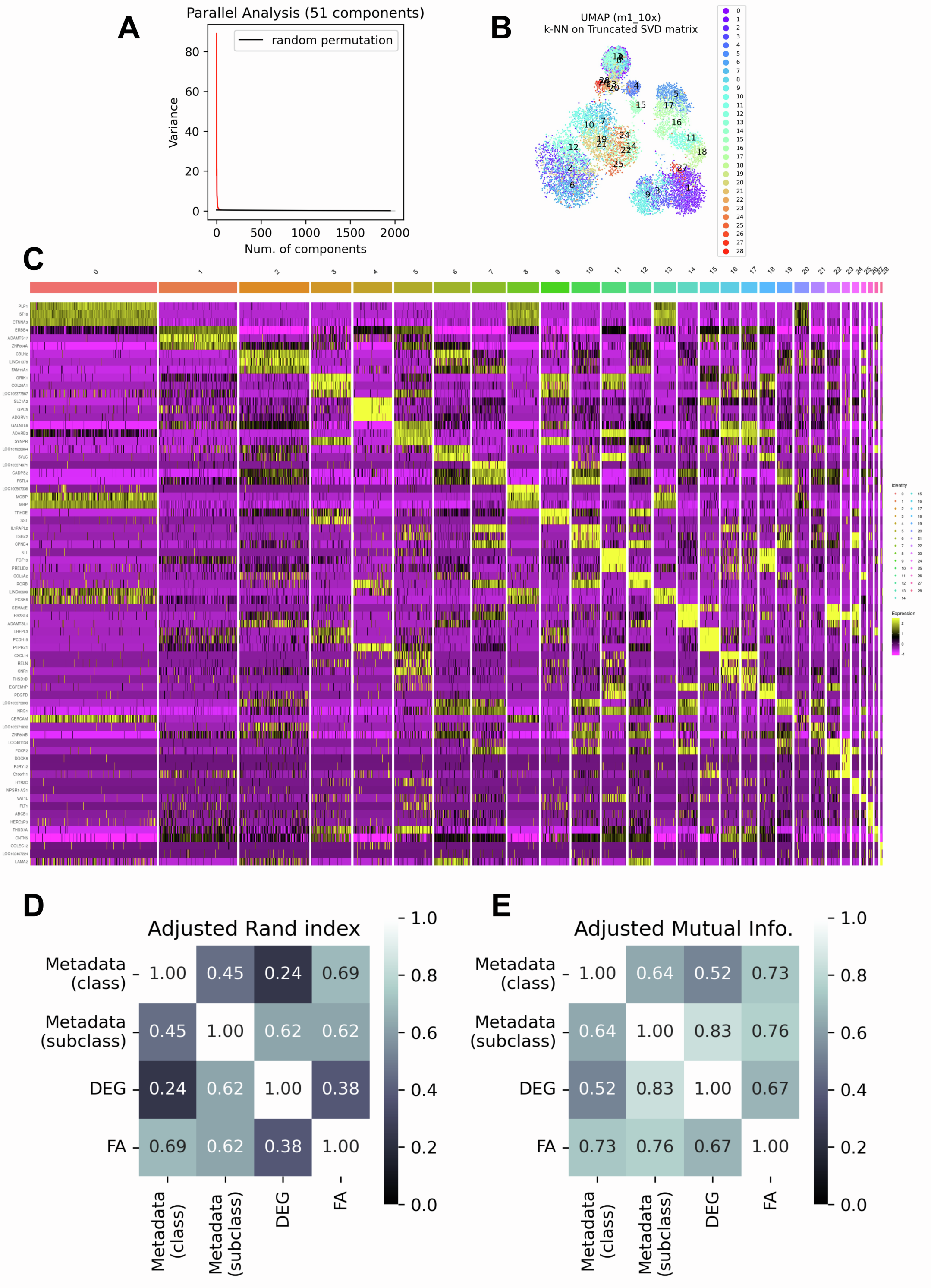
A
Parallel Analysis (51 components)
random permutation
Variance
Num. of components
0
20
40
60
80
500
1000
1500
2000
B
UMAP (m1_10x)
k-NN on Truncated SVD matrix
C
D
Adjusted Rand index
Metadata (class)
Metadata (subclass)
DEG
FA
1.00 0.45 0.24 0.69
0.45 1.00 0.62 0.62
0.24 0.62 1.00 0.38
0.69 0.62 0.38 1.00
0.0
0.2
0.4
0.6
0.8
1.0
E
Adjusted Mutual Info.
Metadata (class)
Metadata (subclass)
DEG
FA
1.00 0.64 0.52 0.73
0.64 1.00 0.83 0.76
0.52 0.83 1.00 0.67
0.73 0.76 0.67 1.00
0.0
0.2
0.4
0.6
0.8
1.0

Figure S5

Figure S5. Conventional workflow for scRNA-seq data processing in m1_10x.

(A) Parallel analyses used to determine the dimensionalities of variable features in m1_10x.

(B) Results of SNN clustering in UMAP.

(C) Heatmaps of the DEGs for respective clusters. The Wilcoxon Rank Sum test was performed to identify DEGs in m1_10x. The top three genes with the largest log FC values were shown for each cluster.

(D–E) Performance comparison between the conventional method and our proposed method (factor analysis + k-means). (D) ARI and (E) AMI were calculated from comparisons of the class labels (cell type information) and subclass labels in the metadata of m1_10x.

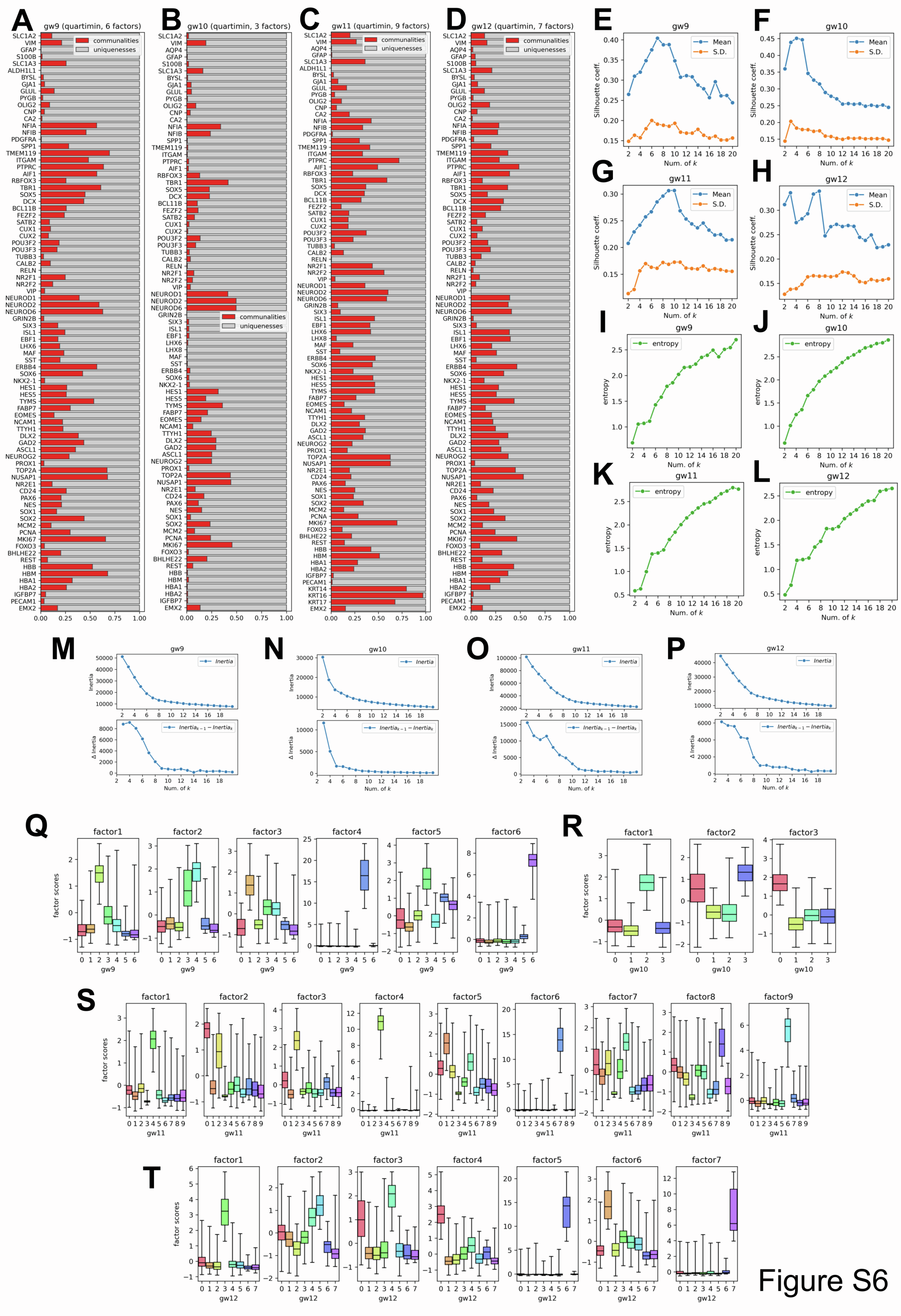
A
gw9 (quartimin, 6 factors)
B
gw10 (quartimin, 3 factors)
C
gw11 (quartimin, 9 factors)
D
gw12 (quartimin, 7 factors)
communalities
uniquenesses
E
gw9
F
gw10
G
gw11
H
gw12
Silhouette coeff.
Mean
S.D.
Num. of k
I
gw9
J
gw10
K
gw11
L
gw12
entropy
M
gw9
N
gw10
O
gw11
P
gw12
Inertia
Δ Inertia
Num. of k
Q
R
S
T
factor scores
factor1
factor2
factor3
factor4
factor5
factor6
factor7
factor8
factor9

Figure S6

Figure S6. Supplemental information for factor analysis + k-means clustering in GSE265388.

(A–D) The communalities and the uniquenesses for each variable in the models.

(E–H) Transition of the mean and S.D. for the silhouette coefficients.

(I–L) Transition of entropies for categorical distributions in which parameters were point-estimated using the sample size ratios of clusters.

(M–P) The elbow plots and the differences of inertia ($\Delta Inertia_k \coloneqq Inertia_{k-1} - Inertia_k$).

(Q–T) Coordinates of clusters (corresponding to factors) in each axis.

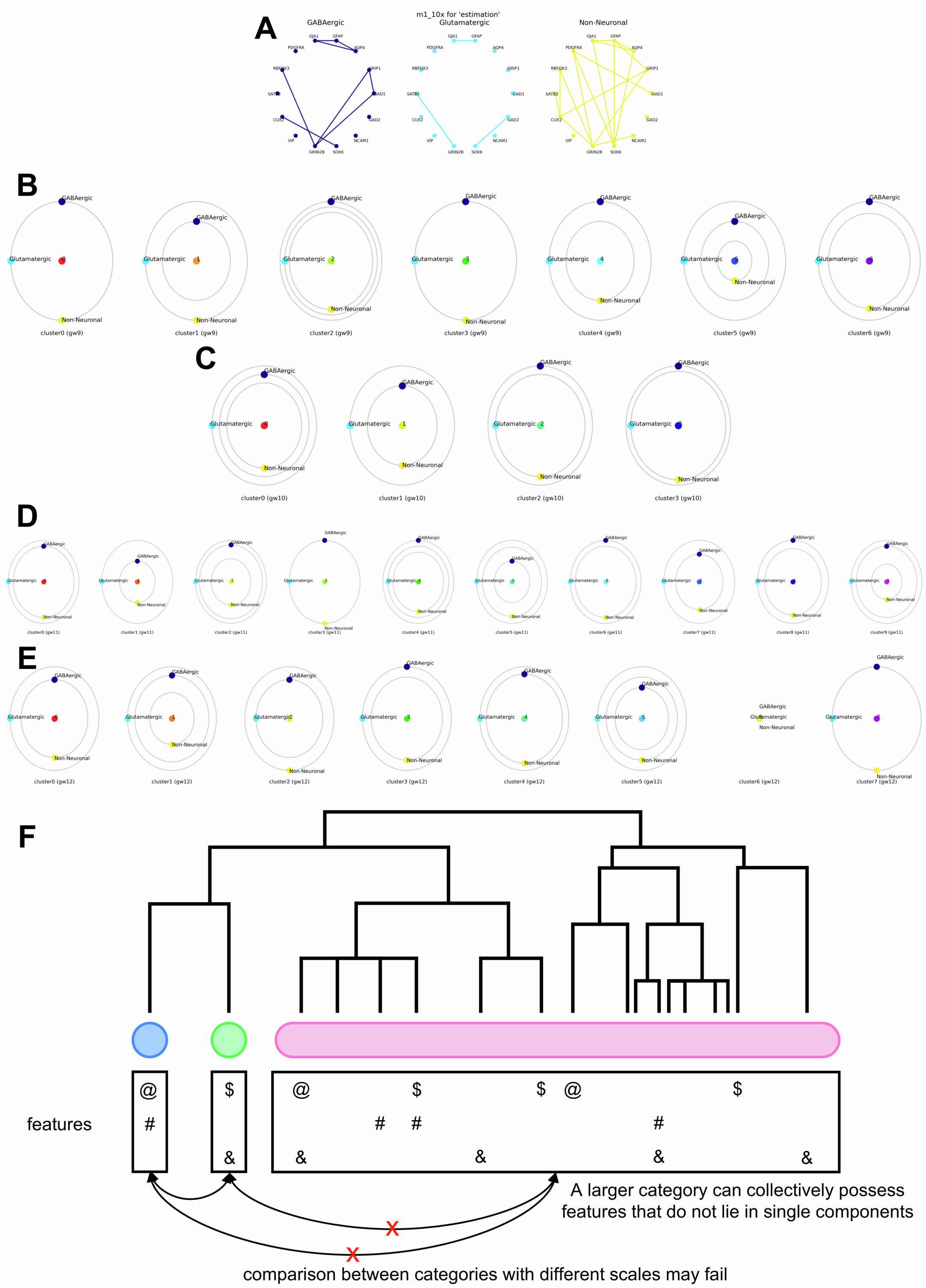
A
m1_10x for 'estimation'
GABAergic
Glutamatergic
Non-Neuronal
GJA1 GFAP PDGFRA AQP4 RBFOX3 GRIP1 SATB2 GAD1 CUX2 GAD2 VIP NCAM1 GRIN2B SOX6
B
cluster0 (gw9) cluster1 (gw9) cluster2 (gw9) cluster3 (gw9) cluster4 (gw9) cluster5 (gw9) cluster6 (gw9)
C
cluster0 (gw10) cluster1 (gw10) cluster2 (gw10) cluster3 (gw10)
D
cluster0 (gw11) cluster1 (gw11) cluster2 (gw11) cluster3 (gw11) cluster4 (gw11) cluster5 (gw11) cluster6 (gw11) cluster7 (gw11) cluster8 (gw11) cluster9 (gw11)
E
cluster0 (gw12) cluster1 (gw12) cluster2 (gw12) cluster3 (gw12) cluster4 (gw12) cluster5 (gw12) cluster6 (gw12) cluster7 (gw12)
F
features
@ # $ &
A larger category can collectively possess
features that do not lie in single components
comparison between categories with different scales may fail

Figure S7

Figure S7. Supplemental information for GRN-based annotation in GSE165388.

(A) GRNs of cell types in m1_10x to be used in estimation (as referential data).

(B–E) Planet plots to show the estimation results.

(F) Demonstration that comparisons between differently scaled categories can fail.
